# Chemokine Receptor Ccr1 Drives Neutrophil-Mediated Kidney Immunopathology and Mortality in Invasive Candidiasis

**DOI:** 10.1371/journal.ppat.1002865

**Published:** 2012-08-16

**Authors:** Michail S. Lionakis, Brett G. Fischer, Jean K. Lim, Muthulekha Swamydas, Wuzhou Wan, Chyi-Chia Richard Lee, Jeffrey I. Cohen, Phillip Scheinberg, Ji-Liang Gao, Philip M. Murphy

**Affiliations:** 1 Clinical Mycology Unit, Laboratory of Molecular Immunology, National Institute of Allergy and Infectious Diseases (NIAID), National Institutes of Health (NIH), Bethesda, Maryland, United States of America; 2 Molecular Signaling Section, Laboratory of Molecular Immunology, NIAID, National Institutes of Health (NIH), Bethesda, Maryland, United States of America; 3 Laboratory of Pathology, Center for Cancer Research, National Cancer Institute (NCI), National Institutes of Health (NIH), Bethesda, Maryland, United States of America; 4 Medical Virology Section, Laboratory of Infectious Diseases, National Institute of Allergy and Infectious Diseases (NIAID). National Institutes of Health (NIH), Bethesda, Maryland, United States of America; 5 Hematology Branch, National Heart, Lung and Blood Institute (NHLBI), National Institutes of Health (NIH), Bethesda, Maryland, United States of America; Carnegie Mellon University, United States of America

## Abstract

Invasive candidiasis is the 4^th^ leading cause of nosocomial bloodstream infection in the US with mortality that exceeds 40% despite administration of antifungal therapy; neutropenia is a major risk factor for poor outcome after invasive candidiasis. In a fatal mouse model of invasive candidiasis that mimics human bloodstream-derived invasive candidiasis, the most highly infected organ is the kidney and neutrophils are the major cellular mediators of host defense; however, factors regulating neutrophil recruitment have not been previously defined. Here we show that mice lacking chemokine receptor Ccr1, which is widely expressed on leukocytes, had selectively impaired accumulation of neutrophils in the kidney limited to the late phase of the time course of the model; surprisingly, this was associated with improved renal function and survival without affecting tissue fungal burden. Consistent with this, neutrophils from wild-type mice in blood and kidney switched from Ccr1^lo^ to Ccr1^high^ at late time-points post-infection, when Ccr1 ligands were produced at high levels in the kidney and were chemotactic for kidney neutrophils *ex vivo*. Further, when a 1∶1 mixture of *Ccr1^+/+^* and *Ccr1^−/−^* donor neutrophils was adoptively transferred intravenously into *Candida*-infected *Ccr1^+/+^* recipient mice, neutrophil trafficking into the kidney was significantly skewed toward *Ccr1^+/+^* cells. Thus, neutrophil Ccr1 amplifies late renal immunopathology and increases mortality in invasive candidiasis by mediating excessive recruitment of neutrophils from the blood to the target organ.

## Author Summary

Invasive infection by the yeast *Candida* represents a significant cause of morbidity and mortality in patients in the intensive care unit. Neutrophils, which are recruited to sites of *Candida* infection by chemokines and their receptors, are important immune cells in host defense against invasive candidiasis. Consistent with that, lack of neutrophils is a well-established risk factor for adverse outcome after infection. In this study, we performed a broad survey of the chemokine system in a mouse model of invasive candidiasis with an aim to determine factors that regulate neutrophil trafficking to sites of infection. We used that survey to identify Ccr1 as a mediator of mortality in the model via excessive recruitment of neutrophils from the blood to the kidney that results in kidney tissue injury. Strikingly, the effect of Ccr1 on neutrophil accumulation in the kidney was not seen until the late phase of the infection, when the receptor was up-regulated on the neutrophil surface. Together these data demonstrate that neutrophils, besides their recognized protective roles in antifungal host defense, may also exert detrimental effects by causing uncontrolled tissue damage, and identify Ccr1 as a mediator of neutrophil tissue injury in a mouse model of invasive candidiasis.

## Introduction

Invasive candidiasis, mainly caused by the opportunistic yeast *Candida albicans*, is the fourth leading cause of nosocomial bloodstream infection in the US and worldwide [1]. The annual incidence of invasive candidiasis has dramatically increased over the past decades and is now estimated at >20 cases per 100,000 population (>60,000 cases/year in the US) with an associated annual cost of two billion dollars in the US [1]–[3]. Vaccines are not available and despite administration of potent antifungal treatment, mortality of invasive candidiasis patients exceeds 40% [1], [4]. Thus, identification of novel therapeutic targets based on better understanding of its molecular pathogenesis is needed.

A mouse model has been used extensively to study invasive candidiasis. In the model, mice develop renal failure and septic shock [5], similar to humans with invasive candidiasis [4], and the kidney is the primary target organ [5], [6]. Several studies in this model have established the importance of innate immunity in anti-*Candida* host defense. Neutrophils and monocytes/macrophages are the predominant leukocytes accumulating in *Candida*-infected tissues in both mice and humans [7], [8]. *In vivo* depletion of neutrophils or macrophages using anti-Gr1 monoclonal antibody or clodronate-loaded liposomes respectively, results in increased tissue fungal burden and accelerated mortality [9], [10]. In contrast, mice with severe combined immunodeficiency that lack functional lymphocytes do not have increased mortality after invasive candidiasis [11]. In agreement with the requirement of innate over adaptive immunity for host defense against invasive candidiasis, patients with chronic granulomatous disease but not those with HIV infection are at heightened risk for invasive candidiasis [12].

At the molecular level, little information is available regarding the factors regulating phagocyte recruitment to *Candida*-infected organs. Here, we have defined the profile of chemoattractants and chemoattractant receptors induced by *Candida* in infected tissues of mice. We have used this profile to identify the chemokine receptor Ccr1 as a candidate, and have tested its functional importance genetically using Ccr1-deficient mice. Our data show that Ccr1 plays a pathogenic role in invasive candidiasis by mediating renal immunopathology via excessive neutrophil recruitment from the blood into the kidney.

## Results

### Invasive candidiasis induces expression of leukocyte chemotactic factors in infected organs

**Figure 1:**
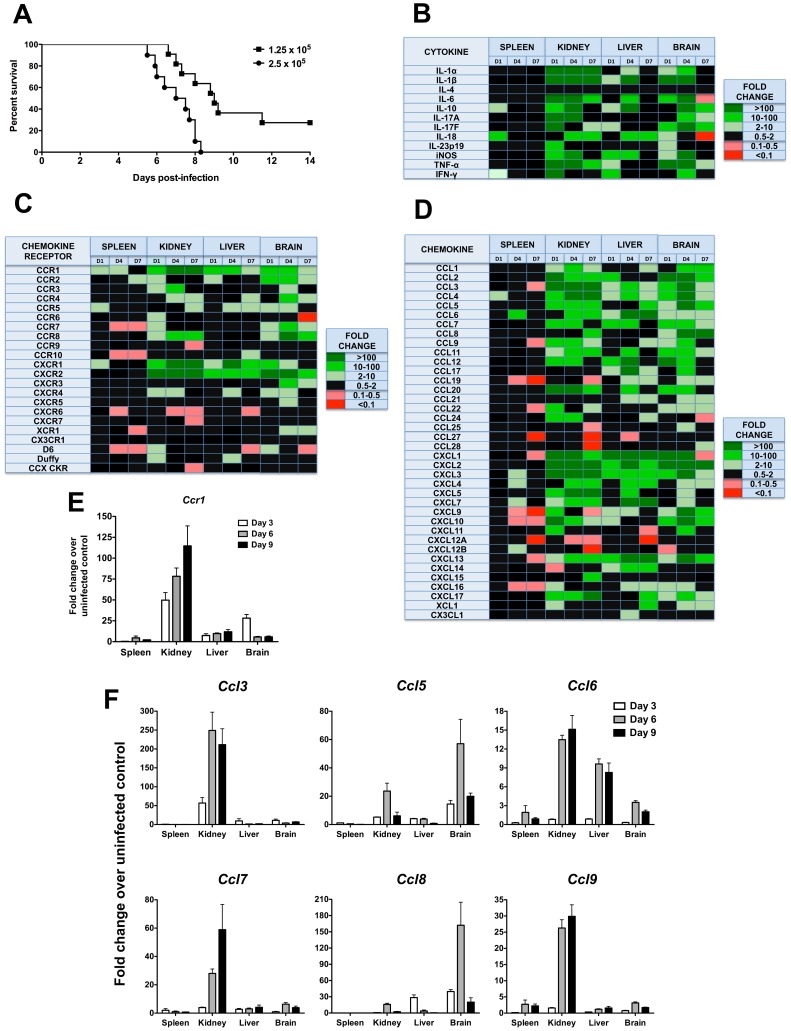
Phagocyte-targeted chemokines and their receptors are induced in a mouse model of invasive candidiasis. (A) Mortality of mice intravenously injected with *Candida albicans* is inoculum-dependent. The inocula tested in this study are given in the upper right hand corner of the figure as CFU. (B–D) Transcriptional profile of (B) cytokines, (C) chemokine receptors and (D) chemokines in a mouse model of invasive candidiasis. D1, day 1; D4, day 4; D7, day 7 post-infection. Color code is indicated to the right of each panel. Black color denotes either 0.5–2.0-fold change in gene expression over uninfected control mice, or a larger change that did not reach statistical significance. Inoculum, 2.5×10^5^ CFU. Data are from one experiment with three animals per time point. (E) Ccr1 and (F) its ligands Ccl3, Ccl5, Ccl6, Ccl7, Ccl8 and Ccl9 are induced after *Candida* infection, predominantly in the kidney. Inoculum, 1.25×10^5^ CFU. Data are from one experiment with four animals per time point.

To identify molecules that might be important in the pathogenesis of invasive candidiasis, we screened 72 immunomodulatory factors (22 chemokine receptors, 38 chemokines, 12 cytokines) over time for differential mRNA expression in the kidney, spleen, liver and brain of uninfected mice versus in mice injected with 2.5×10^5^ CFU, an inoculum that results in 100% mortality by day 8 post-infection (Figure 1A). Twelve of the 22 chemokine receptors, 31 of the 38 chemokines, and 11 of the 12 cytokines were induced ≥2-fold in at least two different organs of all mice examined (Figure 1B, 1C and 1D). Of the cytokines induced (Figure 1B), IL-1α, IL-1β, IL-6, IL-10, IL-17A, IL-18, IFN-γ, and TNF-α have been previously shown to play an important role in the pathogenesis of invasive candidiasis *in vivo* [13]–[21].

Of the chemoattractant mediators, several chemokines (Ccl25, Ccl27, Ccl28) and chemokine receptors (Ccr9, Ccr10, Cxcr5, Cxcr6, Cxcr7) associated with adaptive immunity were not induced after *Candida* infection (Figure 1C and 1D). In contrast, several chemoattractants associated with the innate immune response were significantly induced. Specifically, with regard to candidate chemotactic systems that might guide leukocyte accumulation in *Candida*-infected organs our data point to the phagocyte-targeted CC chemokines Ccl2, Ccl3, Ccl4, Ccl5 and Ccl7 and their receptors Ccr1, Ccr2 and Ccr5, and to the ELR CXC chemokines Cxcl1 (KC), Cxcl2 (MIP-2), Cxcl5 and Cxcl7 and their receptors Cxcr1 and Cxcr2 (Figure 1C and 1D). For the remainder of this paper, we focus our analysis on the role of Ccr1 in the pathogenesis of invasive candidiasis, as Ccr1 and its ligands were among the phagocyte-targeted chemotactic factors that were most highly induced in the model.

### Lack of Ccr1 results in increased survival without impairing anti-*Candida* control

In separate experiments, induction of Ccr1 and its ligands Ccl3, Ccl5, Ccl6, Ccl7, Ccl8 and Ccl9 by invasive candidiasis was examined in greater detail in mice injected with 1.25×10^5^ CFU of *C. albicans*, which causes ∼80% mortality by day 14 post-infection (Figure 1A). As shown in Figure 1E, Ccr1 induction was greatest in the kidney, peaking at a >100-fold increase over the uninfected state on day 9 post-infection. Ccr1 was induced to a lesser extent in the liver throughout the course of the infection and in the brain more transiently peaking on day 3. In contrast, weak induction was observed in the spleen. The Ccr1 ligands were all induced with variable dynamics; Ccl5 and Ccl8 were induced most prominently in the brain, the others most predominantly in the kidney (Figure 1F).

**Figure 2:**
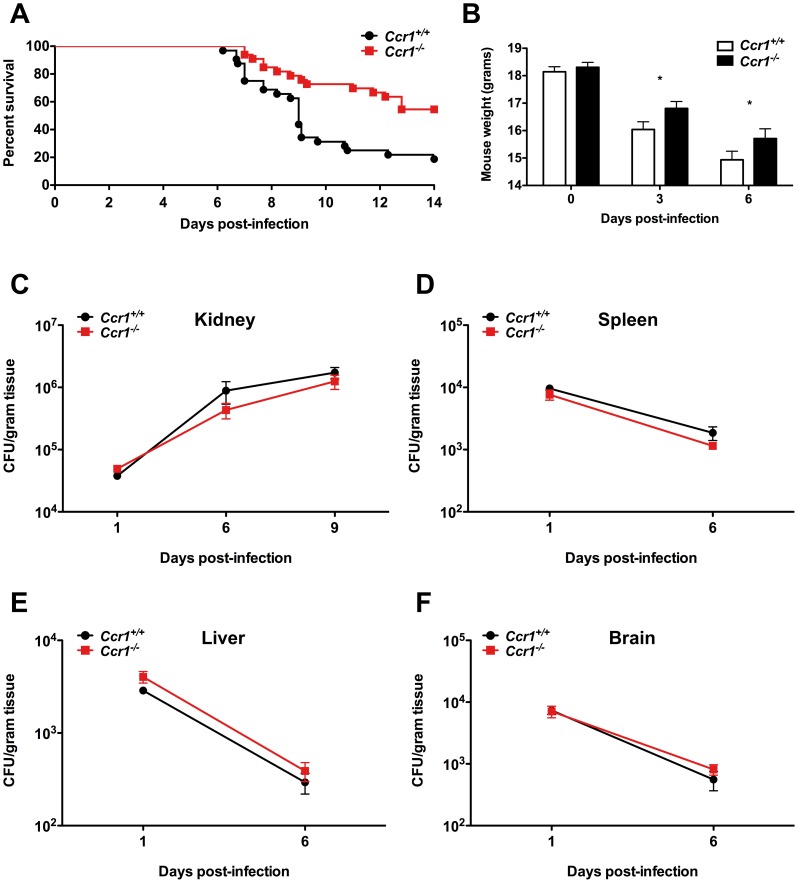
Lack of Ccr1 improves survival without affecting tissue fungal burden in a mouse model of invasive candidiasis. (A) *Ccr1^−/−^* mice have 3-fold better survival compared to *Ccr1^+/+^* mice. *P* = 0.0008. Data are from three independent experiments with a total of thirty-two *Ccr1^+/+^* and thirty-three *Ccr1^−/−^* mice. (B) *Ccr1^−/−^* mice develop less severe weight loss compared to *Ccr1^+/+^* mice after *Candida* infection. ^*^ *P*<0.05. Data are from three independent experiments using twenty-four *Ccr1^+/+^* and twenty-one *Ccr1^−/−^* mice. (C–F) Ccr1 deficiency does not affect tissue fungal burden in kidney (C), spleen (D), liver (E) or brain (F). Data are from four independent experiments using twenty-four *Ccr1^+/+^* and twenty-one *Ccr1^−/−^* mice. Inoculum, 1.25×10^5^ CFU.

Because Ccr1 is expressed on neutrophils and monocytes/macrophages [22], which are critical for survival after invasive candidiasis, and since Ccr1 and its ligands were induced in the model, we investigated the *in vivo* role of Ccr1 in the pathogenesis of invasive candidiasis using *Ccr1^−/−^* mice. Surprisingly, *Ccr1^−/−^* mice infected with *Candida* had 3-fold better survival than *Ccr1^+/+^* mice at day 14 post-infection (18% versus 56%; Figure 2A; *P*<0.001), an effect maintained through day 28 post-infection (data not shown; *P* = 0.001), and exhibited less pronounced weight loss (Figure 2B; *P*<0.05). Mortality with this inoculum began at day 6 post-infection and the greatest divergence in infection susceptibility between *Ccr1^+/+^* and *Ccr1^−/−^* mice was seen around day 9 (Figure 2A). Since fungal burden in the kidney has been shown to correlate strongly with mortality in this model [5], [6], we examined tissue fungal burden in *Ccr1^+/+^* and *Ccr1^−/−^* mice at different time-points after infection to determine whether fungal burden differences might account for the difference in survival. As shown in Figure 2C, 2D, 2E and 2F, Ccr1 deficiency did not affect *Candida* tissue burden in the kidney, spleen, liver or brain. Moreover, no differences were observed in the distribution or morphogenic states of *Candida* infiltration between *Ccr1^+/+^* and *Ccr1^−/−^* mice in the kidney, spleen, liver or brain as determined by Periodic-acid Schiff staining (Figure S1A and data not shown). These data demonstrate that Ccr1 deficiency leads to improved survival after invasive candidiasis, which is not related to a differential ability of *Ccr1^+/+^* and *Ccr1^−/−^* mice to control the pathogen burden.

### Ccr1 promotes kidney tissue injury, immunopathology and renal failure after *Candida* infection

**Figure 3:**
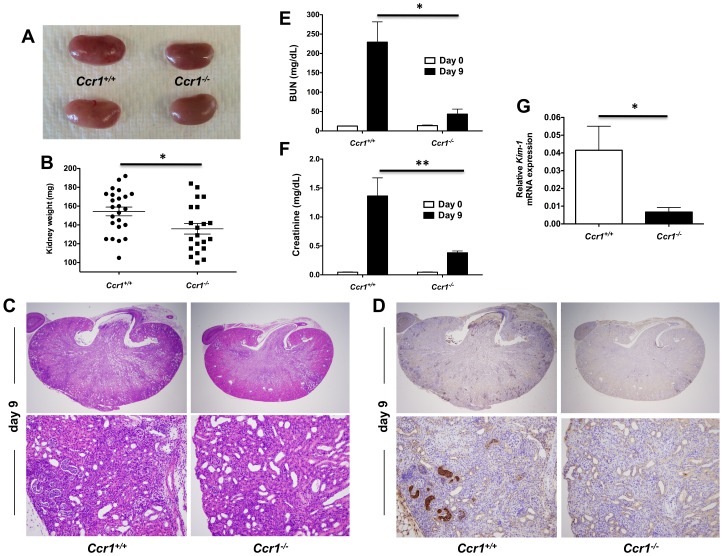
Ccr1 deficiency results in decreased kidney tissue damage. (A) Representative gross pathology pictures of *Ccr1^+/+^* and *Ccr1^−/−^* kidneys at day 9 post-infection. *Ccr1^+/+^* kidneys appear more swollen and pale compared to *Ccr1^−/−^* kidneys. (B) Weight of *Ccr1^−/−^* kidneys is significantly less compared to *Ccr1^+/+^* kidneys at day 9 post-infection. ^*^ *P* = 0.01. Data are from four independent experiments with twenty-four *Ccr1^+/+^* and twenty-one *Ccr1^−/−^* mice. (C) Representative histopathology cross-sections of *Ccr1^+/+^* and *Ccr1^−/−^* kidneys at day 9 post-infection. *Ccr1^+/+^* kidneys sustain significantly greater inflammatory changes compared to *Ccr1^−/−^* kidneys. Original magnifications: 20× (top row) and 400× (bottom row). Images are representative of eight *Ccr1^+/+^* and eight *Ccr1^−/−^* mice from three independent experiments. (D) 7/4 IHC staining of *Ccr1^+/+^* and *Ccr1^−/−^* kidneys at day 9 post-infection. Original magnifications: 20× (top row) and 400× (bottom row). Images are representative of six mice from two independent experiments. (E–F) Kidney failure is significantly more severe in *Ccr1^+/+^* compared to *Ccr1^−/−^* mice at day 9 post-infection as shown by (E) BUN and (F) creatinine measurements in mouse serum. ^*^ *P*<0.001. ^**^ *P*<0.01. Data are from two independent experiments using eight *Ccr1^+/+^* and eight *Ccr1^−/−^* mice. (G) Relative mRNA expression of KIM-1 is greater in *Ccr1^+/+^* kidneys compared to *Ccr1^−/−^* kidneys at day 9 post-infection. Data are from one experiment with four *Ccr1^+/+^* and four *Ccr1^−/−^* mice per time point. ^*^ *P* = 0.01.

We next examined whether the survival difference between infected *Ccr1^+/+^* and *Ccr1^−/−^* mice was due to dysregulated immunopathology. Indeed, *Ccr1^+/+^* kidneys had severe swelling and pallor in gross pathology (Figure 3A) and exhibited a more pronounced increase in weight post-infection compared to *Ccr1^−/−^* kidneys (Figure 3B; *P* = 0.01), a kidney-specific finding not observed in the spleen, liver or brain (Figure S1B). Histopathological analysis revealed that *Ccr1^+/+^* kidneys sustained more extensive tissue damage with larger multifocal areas of abscess formation compared to *Ccr1^−/−^* kidneys (Figure 3C, top row; Figure S1C shows uninfected kidney for comparison). This difference was particularly prominent in the renal cortex; >80% of the surface area was affected by abscess formation in *Ccr1^+/+^* mice, whereas <20% of cortical involvement was observed in *Ccr1^−/−^* mice (Figure 3C, bottom row & Figure S1D; Figure S1E shows uninfected renal cortex for comparison). Of note, no difference in histopathology was observed between *Candida*-infected *Ccr1^+/+^* and *Ccr1^−/−^* mice in the spleen, liver or brain (Figure S1F).

To characterize the cellular composition of the abscesses seen in *Ccr1^+/+^* and *Ccr1^−/−^* kidneys post-infection, we performed immunohistochemistry (IHC) using 7/4, a marker on the surface of mouse neutrophils and monocytes/macrophages [23]. 7/4^+^ cells were more frequent in *Ccr1^+/+^* kidneys than in *Ccr1^−/−^* kidneys post-infection (Figure 3D). Consistent with this, *Ccr1^+/+^* mice developed more severe renal failure after infection (Figure 3E and 3F; *P*<0.01), and *Ccr1^+/+^* kidneys displayed significantly greater expression of kidney injury molecule-1 (KIM-1; Figure 3G; *P* = 0.01), a marker of tubular epithelial damage [24] currently used for diagnosis of acute kidney injury in humans [25]. These data show that Ccr1 deficiency results in attenuated kidney tissue injury, less severe renal failure and decreased phagocyte accumulation and abscess formation in the kidney after *Candida* infection.

### Ccr1 mediates neutrophil-specific accumulation in the kidney only late in the course of invasive candidiasis

**Figure 4:**
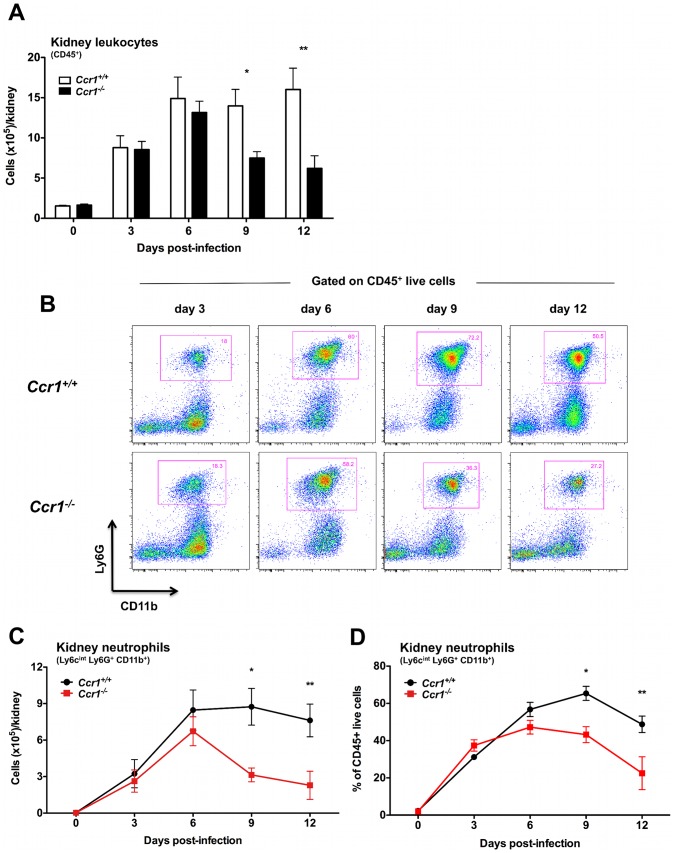
Ccr1 mediates neutrophil accumulation in the kidney at late time-points post-infection. (A) Accumulation of CD45^+^ leukocytes in *Ccr1^+/+^* and *Ccr1^−/−^* kidneys post-*Candida* infection. ^*^ *P* = 0.01. ^**^ *P*<0.01. (B) Representative FACS plots of Ly6c^int^Ly6G^+^CD11b^+^ neutrophils in *Ccr1^+/+^* and *Ccr1^−/−^* kidneys at different time-points after *Candida* infection. (C) Accumulation of Ly6c^int^Ly6G^+^CD11b^+^ neutrophils is Ccr1-mediated late (days 9 and 12) but not early (days 3 and 6) post-infection. ^*^ *P*<0.01. ^**^ *P* = 0.01. (D) Percent of Ly6c^int^Ly6G^+^CD11b^+^ neutrophils in total CD45^+^ leukocytes accumulating in *Ccr1^+/+^* and *Ccr1^−/−^* kidneys at different time-points after *Candida* infection. ^*^ *P*<0.01. ^**^ *P* = 0.02.

To assess quantitatively and temporally the effect of Ccr1 on specific leukocyte accumulation in the model, we harvested leukocytes from the organs and defined their immunophenotypes by FACS on days 3, 6, 9 and 12 post-infection. As previously shown [8], neutrophils were the predominant leukocytes accumulating in the kidney after *Candida* infection, followed by macrophages and dendritic cells. Of interest, whereas no difference in accumulation of CD45^+^ leukocytes was seen up until day 6, *Ccr1^−/−^* kidneys accumulated ∼60% fewer leukocytes compared to *Ccr1^+/+^* kidneys late at days 9 and 12 post-infection (Figure 4A; *P*≤0.01). This time-dependent difference in Ccr1-mediated leukocyte accumulation in the kidney was exclusively accounted for by Ly6c^int^Ly6G^+^CD11b^+^ neutrophils, which accumulated in a Ccr1-independent manner until day 6 post-infection, but thereafter were ∼60% decreased in *Ccr1^−/−^* kidneys (Figure 4B, 4C and 4D; P≤0.01). In contrast, neutrophil accumulation was Ccr1-independent throughout the entire course of invasive candidiasis in the spleen, liver and brain (Figure S2A, S2B and S2C).

No differences were observed between *Ccr1^+/+^* and *Ccr1^−/−^* kidneys post-infection in the accumulation of Ly6c^hi^CD11b^+^ inflammatory monocytes, NK1.1^+^ cells, MHCII^+^F4/80^+^CD11c^−^ macrophages, MHCII^+^CD11c^+^ dendritic cells, CD4^+^ and CD8^+^ T lymphocytes or CD19^+^ B cells (Figure S3A, S3B, S3C, S3D, S3E, S3F and S3G), although there was a trend towards lower B cell numbers in *Ccr1^−/−^* kidneys at days 9 and 12 post-infection (*P* = 0.07). Similarly, Ccr1 deficiency did not significantly affect accumulation of these immune cell types in the spleen, liver or brain (data not shown). These data indicate that Ccr1 is important for neutrophil accumulation in the kidney late but not early in the course of invasive candidiasis, while it is dispensable for neutrophil accumulation in the other organs tested, and for accumulation of other leukocytes in all the organs examined.

### Decreased neutrophil accumulation in *Ccr1^−/−^* kidneys late after invasive candidiasis is not caused by reduced neutrophil survival or increased neutrophil egress into the urine

**Figure 5:**
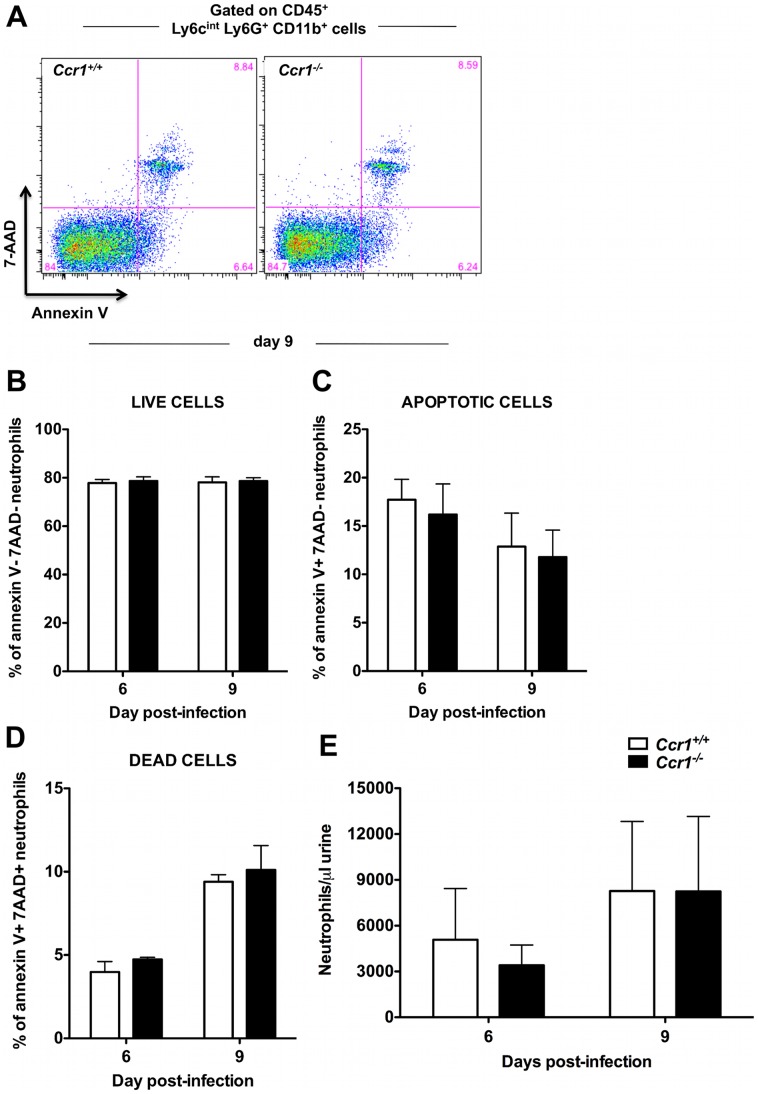
Ccr1 deficiency does not affect kidney neutrophil survival or efflux of neutrophils into the urine. (A) Representative FACS plots of annexin V and 7-AAD staining in kidney neutrophils at day 9 post-infection. Percent of (B) live annexin V*^−^* 7-AAD*^−^*, (C) apoptotic annexin V*^+^* 7-AAD*^−^*, and (D) dead annexin V*^+^* 7-AAD*^+^* kidney neutrophils is similar in *Ccr1^+/+^* and *Ccr1^−/−^* mice at days 6 and 9 post-infection. Data are shown from one of two independent experiments with similar pattern of results using a total of seven *Ccr1^+/+^* and seven *Ccr1^−/−^* mice per time-point. (E) Accumulation of neutrophils in the urine of *Ccr1^+/+^* and *Ccr1^−/−^* mice is similar at days 6 and 9 post-infection. Data are from two experiments with eight to ten *Ccr1^+/+^* and eight to ten *Ccr1^−/−^* animals per time-point.

Because both histopathological and immunophenotypic analyses revealed differences between *Ccr1^+/+^* and *Ccr1^−/−^* mice only in the kidney, in the remainder of our studies we focused on that organ. Thus, we next investigated the mechanism(s) underlying the late Ccr1-dependent phase of neutrophil accumulation in the kidney after invasive candidiasis. Decreased neutrophil accumulation in *Ccr1^−/−^* kidneys could be explained by the net effect of (1) decreased trafficking of neutrophils from the blood into the kidney, and/or (2) decreased survival of neutrophils in *Ccr1^−/−^* mice after entry into the kidney, and/or (3) increased egress of neutrophils from the kidney into the urine. First, we examined the effect of Ccr1 deficiency on neutrophil survival. Chemokine receptors have previously been reported to mediate cell survival signals [26]. However, Ccr1 deficiency did not appear to affect neutrophil survival in the model since *Ccr1^+/+^* and *Ccr1^−/−^* neutrophils had similar levels by FACS of annexin V^−^ 7-AAD^−^ cells (as a measure of viability), annexin V^+^ 7-AAD^−^ cells (as a measure of apoptosis) and annexin V^+^ 7-AAD^+^ cells (as a measure of death) in both the kidney (Figure 5A, 5B, 5C and 5D) and spleen (Figure S4). With regard to egress, it is conceivable that a critical threshold may exist for neutrophils in the kidney beyond which the collecting system and renal pelvis becomes obstructed and neutrophils no longer flow out of the kidney in the urine. This would plausibly be a time-dependent process and could explain the delayed Ccr1 dependence of neutrophil accumulation in the model. However, we observed no difference in the number of neutrophils in the urine of *Ccr1^+/+^* and *Ccr1^−/−^* mice post-infection (Figure 5E). Hence, we found no evidence that decreased neutrophil accumulation in *Ccr1^−/−^* kidneys late after infection was caused by decreased neutrophil survival or increased neutrophil egress into the urine.

### Ccr1 is expressed on blood and kidney neutrophils late in the course of invasive candidiasis

**Figure 6:**
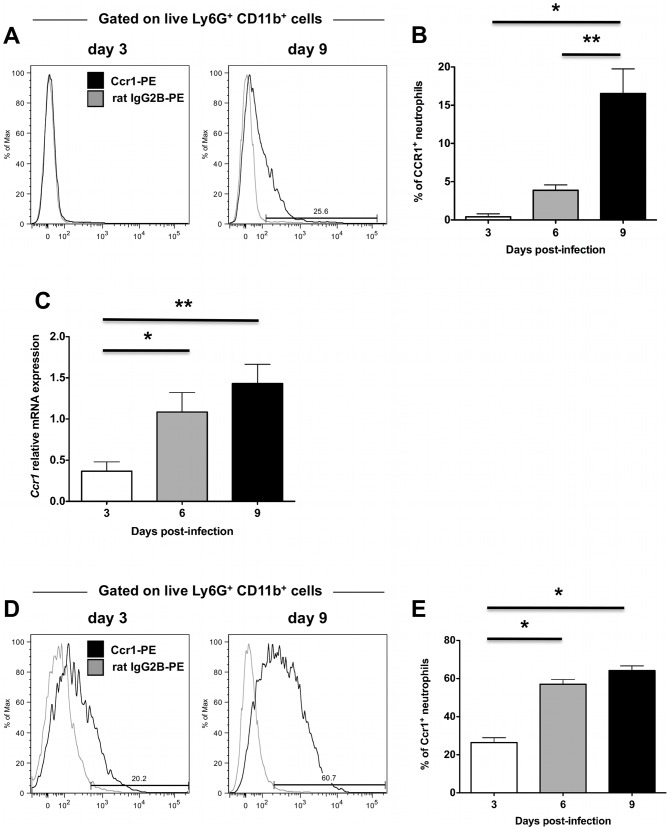
Ccr1 expression increases late after *Candida* infection in blood and kidney neutrophils. (A) Representative FACS histograms of Ccr1 staining on blood neutrophils at days 3 and 9 post-infection. (B) Ccr1 expression on blood neutrophils is significantly increased at day 9 compared to days 3 and 6 post-infection. ^*^ *P*<0.01. ^**^ *P* = 0.03. Data are from two independent experiments using four to ten *Ccr1^+/+^* mice per time-point. (C) Relative Ccr1 mRNA expression in MACS-sorted kidney neutrophils increases late post-infection. Data are from two independent experiments using six to eight *Ccr1^+/+^* mice per time-point. ^*^ *P* = 0.02, ^**^ *P* = 0.003 (D) Representative FACS histograms of Ccr1 staining on kidney neutrophils at days 3 and 9 post-infection. (E) Ccr1 expression on kidney neutrophils is significantly increased at days 6 and 9 compared to day 3 post-infection. ^*^ *P*<0.0001. Data are from three independent experiments using six to eight *Ccr1^+/+^* mice per time-point.

Based on the above findings, we focused next on the third possibility, that Ccr1 mediates neutrophil trafficking from the blood into the kidney, but only in the late phase of the time course of the model. For this to be plausible, (a) the receptor should be expressed on the surface of blood neutrophils (before the cells enter into the kidney) late after infection, and (b) the Ccr1-targeting chemokines should be induced in the kidney at the same time in order to generate a chemokine concentration gradient capable of recruiting Ccr1^+^ blood neutrophils into the kidney via mediating activation, firm adhesion and transmigration of neutrophils on the endothelial surface together with adhesion molecules [22]. Hence, we first looked at the expression of Ccr1 in blood neutrophils of *Ccr1^+/+^* mice during invasive candidiasis. We found that Ccr1 was not detected on blood neutrophils in uninfected mice (data not shown), and was not detected until day 9 post-infection, when ∼20% of neutrophils stained positive (Figure 6A and 6B; *P*<0.05). Further, when MACS-sorted neutrophils from *Ccr1^+/+^* kidneys were tested, Ccr1 mRNA was significantly greater in the late phase (days 6 and 9 post-infection) relative to the early phase (day 3 post-infection) (Figure 6C; *P*<0.05). This was confirmed at the protein level by FACS. In particular, the percentage of Ccr1^+^ kidney neutrophils increased from ∼20–30% on day 3 post-infection to ≥60% on day 9 post-infection (Figure 6D and 6E; *P*<0.0001). In addition to neutrophils, Ccr1 was expressed on other myeloid cells in the kidney (monocytes, macrophages) but not on T or B lymphocytes (Figure S5A, S5B, S5C, S5D and S5E). However, in contrast to neutrophils, Ccr1 expression on the other myeloid cells was constant throughout the course of invasive candidiasis (data not shown). Hence, these data show that Ccr1 expression increases on the surface of neutrophils in the blood and kidney late in the course of invasive candidiasis, when neutrophil accumulation is decreased in *Ccr1^−/−^* kidneys.

### Ccr1 agonists are induced in the kidney after *Candida* infection and are chemotactic for neutrophils *ex vivo*

**Figure 7:**
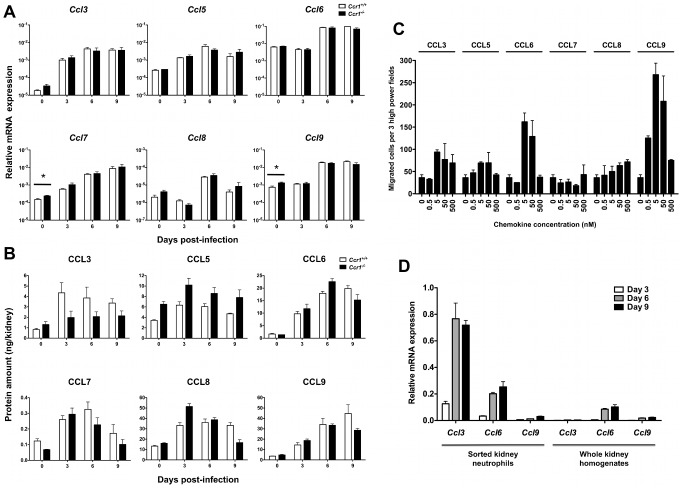
Ccr1 ligands are induced after *Candida* infection and are chemotactic for kidney neutrophils *ex vivo*. (A) Relative mRNA expression of Ccl3, Ccl5, Ccl6, Ccl7, Ccl8 and Ccl9 in *Ccr1^+/+^* and *Ccr1^−/−^* kidneys after infection. Data are shown from one of two independent experiments with similar pattern of results using a total of seven to nine *Ccr1^+/+^* and seven to eight *Ccr1^−/−^* mice per time-point. ^*^ *P* = 0.03 (B) Protein levels of CCL3, CCL5, CCL6, CCL7, CCL8 and CCL9 in *Ccr1^+/+^* and *Ccr1^−/−^* kidneys after infection. Data are from one experiment using four *Ccr1^+/+^* and four *Ccr1^−/−^* mice per time-point. (C) Chemotaxis of MACS-sorted kidney neutrophils recovered from *Ccr1^+/+^* mice at day 9 post-infection to CCL3, CCL5, CCL6, CCL7, CCL8 and CCL9. Data are from one experiment using twelve pooled kidneys from six *Ccr1^+/+^* mice. (D) Relative mRNA expression of Ccl3, Ccl6 and Ccl9 in MACS-sorted *Ccr1^+/+^* kidney neutrophils after *Candida* infection. Data are shown from one of two independent experiments with similar pattern of results using a total of six to eight *Ccr1^+/+^* mice per time-point. Data on relative mRNA expression of the ligands in whole kidney homogenates (right panel) are excerpted from Figure 7A for comparison.

We next determined the levels of the Ccr1 agonists Ccl3, Ccl5, Ccl6, Ccl7, Ccl8 and Ccl9 in *Ccr1^+/+^* and *Ccr1^−/−^* kidneys after infection. Although differential dynamics in induction were observed among them, all Ccr1 ligands were significantly induced at the mRNA (Figure 7A) and protein (Figure 7B) levels in both *Ccr1^+/+^* and *Ccr1^−/−^* kidneys (but not spleen; Figure S6) both early and late after invasive candidiasis. In addition, in order to determine whether neutrophils obtained from *Candida*-infected mice respond to Ccr1 ligands, we sorted neutrophils from kidneys of *Ccr1^+/+^* mice at day 9 post-infection and performed chemotaxis assays with recombinant Ccr1 agonists *ex vivo*. As shown in Figure 7C, five agonists were active with the rank order CCL9>CCL6>CCL3>CCL5>CCL8, whereas CCL7 was inactive. CCL3 has previously been reported to be chemotactic for mouse neutrophils [27], whereas the chemotactic potential of the other Ccr1 agonists has not been previously tested directly for mouse neutrophils.

Next, because both hematopoietic and non-hematopoietic cells have chemokine secreting potential [22], we attempted to define the source of the Ccr1 ligands in the kidney. Due to lack of reliable commercially available antibodies for these chemokines, an IHC approach was not feasible. Instead, we sorted kidney neutrophils during invasive candidiasis in *Ccr1^+/+^* mice and determined the relative mRNA expression for the Ccr1 ligands. As shown in Figure 7D, three were expressed by kidney neutrophils with the rank order Ccl3>Ccl6>Ccl9, whereas Ccl5, Ccl7 and Ccl8 were barely detected or not detected (data not shown). When comparing the relative expression of these chemokines in sorted neutrophils versus whole kidney homogenates, neutrophils appear to be a significant source for Ccl3 and perhaps for Ccl6 and Ccl9 in the kidney after infection, suggestive of a positive feedback loop of neutrophil chemoattraction. These data collectively show that the Ccr1 agonists are induced in the kidney after invasive candidiasis and are chemotactic for kidney neutrophils *ex vivo*.

### Ccr1 mediates neutrophil trafficking from the blood into the kidney after *Candida* infection

**Figure 8:**
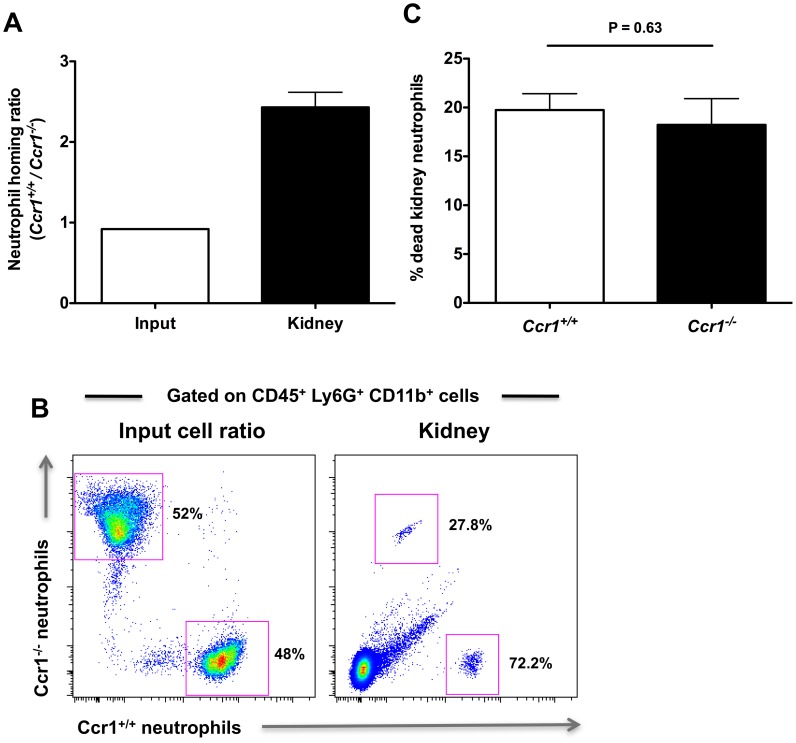
Ccr1 mediates neutrophil trafficking from the blood to the kidney post-infection. Ly6c^int^Ly6G^+^CD11b^+^ neutrophils from *Ccr1^+/+^* and *Ccr1^−/−^* mice were differentially-labeled, mixed at a 1∶1 ratio, and analyzed prior to injection of *Ccr1^+/+^* recipient mice (A and B, left panels). The mixed donor cells were then injected into *Ccr1^+/+^* recipients that had been infected 9 days earlier with *Candida*. The distribution of labeled *Ccr1^+/+^* and *Ccr1^−/−^* donor neutrophils in *Candida*-infected *Ccr1^+/+^* recipient kidneys four hours post-injection is represented as the ratio of *Ccr1^+/+^* to *Ccr1^−/−^* neutrophils in the kidney (A and B, right panels). (C) Labeling of neutrophils does not differentially affect cell survival. Similar percentages of differentially labeled *Ccr1^+/+^* and *Ccr1^−/−^* neutrophils are dead in the kidney four hours after injection. Data are from one experiment using sixteen *Ccr1^+/+^* recipient mice.

To test the role of Ccr1 in neutrophil trafficking from the blood to the kidney directly, we conducted a competitive repopulation study where equal numbers of *Ccr1^+/+^* and *Ccr1^−/−^* Ly6c^int^Ly6G^+^CD11b^+^ neutrophils were isolated by gradient centrifugation from the bone marrows of *Ccr1^+/+^* and *Ccr1^−/−^ Candida*-infected mice at day 9 post-infection, then differentially labeled, mixed 1∶1 and injected into *Candida*-infected *Ccr1^+/+^* recipient mice on day 9 post infection. This time point was chosen because it is the time when (1) neutrophil accumulation in the kidney is Ccr1-dependent in the model (Figure 4B, 4C and 4D), (2) Ccr1 is expressed on the surface of neutrophils in the blood (Figure 6A and 6B), kidney (Figure 6C, 6D and 6E) and bone marrow (data not shown), and (3) Ccr1 ligands are produced in the kidney and are chemotactic for neutrophils (Figure 7A, 7B and 7C). Four hours after neutrophil transfer, mice were euthanized and kidneys were analyzed for the presence of labeled neutrophils. As shown in Figure 8A and 8B, the ratio of labeled *Ccr1^+/+^*:*Ccr1^−/−^* neutrophils prior to injection was approximately 1∶1, as intended. Four hours post-injection, the ratio of *Ccr1^+/+^*:*Ccr1^−/−^* neutrophils in the kidney was strongly skewed towards *Ccr1^+/+^* neutrophils at approximately a 2.5∶1 ratio; this skewing was seen in all 16 mice tested (range: 1.7–4.1). The possibility that the labeling dyes had a differential effect on the survival of *Ccr1^+/+^* versus *Ccr1^−/−^* neutrophils was excluded by FACS, which showed that the proportion of differentially labeled dead *Ccr1^+/+^* and *Ccr1^−/−^* neutrophils was similar (Figure 8C). Therefore, these data indicate that Ccr1 on neutrophils directly mediates neutrophil trafficking from the blood into the kidney late in the course of invasive candidiasis.

### Ccr1 deficiency does not affect the expression of other neutrophil-targeted chemotactic factors or adhesion molecules in the kidney after *Candida* infection

**Figure 9:**
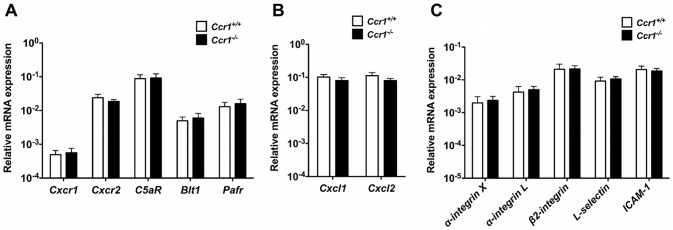
Ccr1 deficiency does not decrease the expression of other neutrophil-targeted chemotactic factors or adhesion molecules in *Candida*-infected kidneys. (A) Relative mRNA expression of neutrophil-targeted chemoattractant receptors in *Ccr1^+/+^* and *Ccr1^−/−^* kidneys at day 9 after infection. (B) Relative mRNA expression of neutrophil-targeted ELR CXC chemokines in *Ccr1^+/+^* and *Ccr1^−/−^* kidneys at day 9 after infection. (C) Relative mRNA expression of neutrophil-targeted adhesion molecules in *Ccr1^+/+^* and *Ccr1^−/−^* kidneys at day 9 after infection. Data are from two independent experiments using nine *Ccr1^+/+^* and eight *Ccr1^−/−^* mice.

Next, we aimed to exclude the possibility that decreased accumulation of neutrophils in *Ccr1^−/−^* kidneys late after infection was caused by decreased expression of other neutrophil-targeted chemoattractant receptors and ligands and/or adhesion molecules in *Ccr1^−/−^* kidneys. For that, we determined the expression level of five major neutrophil-targeted chemoattractant receptors (i.e., the chemokine receptors Cxcr1 and Cxcr2, the complement C5a receptor [C5aR], the leukotriene B4 receptor Blt1, and the platelet activating factor receptor [Pafr]), two major neutrophil-targeted chemokines (i.e., the ELR CXC chemokines Cxcl1 [KC] and Cxcl2 [MIP-2]), and five adhesion molecules (i.e., α-integrin X, α-integrin L, β2-integrin, L-selectin and intercellular adhesion molecule 1 [ICAM-1]) in *Ccr1^+/+^* and *Ccr1^−/−^* kidneys at days 3, 6 and 9 post-infection. We found no significant differences in the expression of these factors between *Ccr1^+/+^* and *Ccr1^−/−^* kidneys at day 6 (Figure S7D, S7E and S7F) or 9 (Figure 9A, 9B and 9C) post-infection. Of the 12 factors examined, a difference in mRNA expression between the two strains was observed at day 3 post-infection only for two: β2-integrin and L-selectin. For both results, mRNA was increased slightly in the *Ccr1^−/−^* kidneys (Figure S7A, S7B and S7C). These data do not provide clear evidence that neutrophil accumulation in the model late after infection is associated with Ccr1-dependent expression of neutrophil-targeted chemotactic factors or adhesion molecules. The differences observed on day 3 post-infection could have a long-term effect but will require additional experimentation to establish any functional significance.

### Ccr1 expression on kidney neutrophils does not affect their immunopathogenic potential or anti-*Candida* effector function

Last, we sought to determine whether the higher Ccr1 expression seen on kidney neutrophils late after infection is associated with an increased ability of neutrophils to cause tissue injury. For that, we sorted Ccr1^lo^ kidney neutrophils at day 3 and Ccr1^high^ kidney neutrophils at day 9 after infection from *Ccr1^+/+^ Candida*-infected mice and compared their potential for degranulation (beta-glucuronidase assay) and oxidative burst (dihydrorhodamine 123 [DHR] assay) *ex vivo*. As shown in Figure S8A, Ccr1^lo^ and Ccr1^high^ kidney neutrophils exhibited no difference in degranulation measured as beta-glucuronidase release. Similarly, we found no difference between Ccr1^lo^ and Ccr1^high^ kidney neutrophils in oxidative burst, both at rest and following stimulation with phorbol myristate acetate (PMA; Figure S8B). Last, Ccr1^lo^ and Ccr1^high^ kidney neutrophils did not exhibit significant differences in anti-*Candida* effector function as demonstrated by their similar capacity for phagocytosis (Figure S8C) and killing (Figure S8D) against *Candida ex vivo*. These data show that the magnitude of Ccr1 expression on kidney neutrophils after *Candida* infection does not appear to directly influence the immunopathogenic potential of neutrophils or their anti-*Candida* effector function in the kidney.

## Discussion

In the present study we demonstrate that the chemokine receptor Ccr1 is associated with fatal neutrophil-mediated immunopathology selectively in the kidney in a mouse model of systemic *Candida albicans* infection. The receptor appears to act directly by promoting neutrophil trafficking from the blood into the kidney, but not until the late phase of the infection when it is turned on selectively by blood neutrophils and when most of its ligands Ccl3, Ccl5, Ccl6, Ccl7, Ccl8 and Ccl9 are expressed at high levels in the kidney. Our conclusions are based on detailed analysis of differences in clinical, pathological, microbiological, immunological and molecular parameters in the model between *Ccr1^+/+^* and *Ccr1^−/−^* mice. Our study is the first identification of a specific chemoattractant receptor that mediates neutrophil trafficking into the kidney and drives neutrophil-mediated immunopathology and mortality in the mouse model of invasive candidiasis. Moreover, the conclusions regarding mechanism are based on a formal *in vivo* neutrophil trafficking study.

We focused on Ccr1 because phagocytes are known to be the main cellular mediators of the immune response in invasive candidiasis [9], [10], and because our broad survey of the chemokine system in the model showed that phagocyte-targeted chemokines and their receptors, including Ccr1 and its ligands, were among the most highly up-regulated. Ccr1 is a chemokine receptor whose functional importance for neutrophils has been more apparent in mice than in humans [27]–[30], and it is also expressed on other hematopoietic and some non-hematopoietic cell types [22].

The outcome in any infectious disease is determined by a balance between host and pathogen factors. In our study, we found several lines of evidence supporting excessive neutrophil accumulation in the kidney resulting in neutrophil-mediated immunopathology as the underlying mechanism for Ccr1-mediated decreased survival in the model. First, Ccr1 deficiency did not affect tissue *Candida* proliferation, an established correlate of survival in previous studies in the model of invasive candidiasis [5], [6], [8]. Second, Ccr1 deficiency was associated with markedly decreased destructive inflammatory changes and tissue injury in the kidney; this was accompanied by less severe kidney failure in *Ccr1^−/−^* mice, which is a major determinant of survival in the model [5]. Third, the decreased kidney tissue injury was associated with a large and selective reduction in neutrophil accumulation in *Ccr1^−/−^* kidneys after infection.

Neutrophils are key innate immune effector cells that play a critical role in phagocytosis and killing of *Candida albicans* [31]. In agreement, neutropenia is a major risk factor for mortality after invasive candidiasis in both mice and humans [9], [32]. Early neutrophil availability in particular has been shown to be critical for protection against invasive candidiasis in mice, as neutrophil depletion within the first 24 hours, but not at later time points post-infection, led to accelerated *Candida* growth and mortality [33]. On the other hand, neutrophils may exert detrimental effects on the host by mediating tissue injury. For example, patients with invasive candidiasis often require administration of corticosteroid therapy after recovery from neutropenia for amelioration of clinical symptoms and attenuation of exuberant inflammation [7]. A similar immune reconstitution inflammatory syndrome has also been described following neutrophil recovery in patients with invasive aspergillosis [34]. In mice, the pathogenic role of neutrophils in invasive candidiasis has been previously suggested by neutrophil depletion studies [33], in which rendering mice neutropenic at late time points after infection when inflammation is pronounced [8], markedly improved survival, presumably due to attenuated tissue injury. Our study is the first to identify a single molecular factor, Ccr1, as one of the mediators of immunopathology in the mouse model of invasive candidiasis.

Ccr1 has been shown to exert both pathogenic and protective effects depending on the inflammatory milieu. For example, Ccr1 was critical for effective host defense against *Toxoplasma gondii*, pneumovirus of mice and *Aspergillus fumigatus* by mediating neutrophil accumulation in the target infection organs [27], [35], [36]. Conversely, Ccr1-mediated neutrophil accumulation was pathogenic in a model of acute respiratory distress syndrome following pancreatitis and a model of sepsis following bacterial peritonitis [28], [29]. The role of Ccr1 has also been studied in the context of renal inflammation. Specifically, Ccr1 deficiency decreased neutrophil and macrophage accumulation in the kidney after ischemia-reperfusion injury, but it had no effect on kidney function or survival [37]. Moreover, Ccr1 deficiency decreased kidney injury in models of Alport disease, focal segmental glomerulosclerosis, unilateral ureteral obstruction and toxic nephropathy; however, the attenuated tissue damage was associated with decreased infiltration of lymphocytes and macrophages but not neutrophils in the kidney [38]–[42].

The mechanism of Ccr1-mediated neutrophil accumulation and immunopathology in the kidney in invasive candidiasis appears to lie at the level of neutrophil trafficking from the blood into the kidney, as shown by our competitive repopulation study using *Ccr1^+/+^* and *Ccr1^−/−^* neutrophils. Additional work using gene-deficient mice and/or antibody depleting strategies will be needed to define which of the Ccr1 ligands, either alone or in synergy, mediate neutrophil trafficking into *Candida*-infected kidneys. Also, because Ccr1 is expressed on neutrophil precursors and mature neutrophils in the bone marrow [27], the impact of Ccr1 deficiency in neutrophil precursor production and egress of neutrophils from the bone marrow to the blood after *Candida* infection merits further investigation. Of note, although Ccr1 is expressed on several immune cell types [22], the reduction in leukocyte accumulation in *Ccr1^−/−^* kidneys was specific to neutrophils. In addition, neutrophil accumulation was Ccr1-dependent only in the kidney but not in the other organs examined, further attesting to the organ-specific nature of the innate immune response in invasive candidiasis [8].

Of interest, Ccr1 mediated neutrophil accumulation in the kidney only late in the course of invasive candidiasis. Three important new questions arise from this finding: the first relates to the signals responsible for Ccr1 induction on neutrophils late but not early in the course of invasive candidiasis. Factors that have been reported to induce Ccr1 on the surface of mouse and human neutrophils include TNF-α, GM-CSF and IFN-γ [43], [44], which were not found to be different between days 3 and 9 in blood or kidney in our model (data not shown). *Candida albicans* grows both in the yeast and hyphal forms in the kidney starting immediately post-infection [8], but unmasking of β-glucan, the pathogen-associated molecular pattern for dectin-1 [45], does not occur until late in the course of invasive candidiasis [46]. Hence, it is plausible that availability of soluble or membrane-bound β-glucan late after invasive candidiasis may contribute to Ccr1 induction on neutrophils.

The second important new question that arises from our study pertains to the chemotactic factors responsible for neutrophil recruitment in the kidney during the first 6 days after infection, when Ccr1 is dispensable for neutrophil accumulation in the kidney. Other neutrophil chemokine receptors such as Cxcr1 and Cxcr2, which were also induced in the model, may play such a role. In this regard, a sequential role for the leukotriene B_4_ receptor Blt1, Ccr1 and Cxcr2 has previously been reported for neutrophil recruitment in a mouse model of arthritis [47]. Because neutrophil availability during the early phase after infection is critical for survival [33], identifying the chemoattractant receptors that mediate trafficking into the kidney early in the course of the infection may reveal novel protective molecular factors against invasive candidiasis in the model. In fact, Cxcr2-deficient mice were previously reported to have impaired ability to control *Candida* proliferation in the kidney and other organs after invasive candidiasis, but accumulation of neutrophils in these tissues was not defined in that study [48].

The third important new question arises from our data that neutrophil accumulation was decreased but not entirely eliminated in *Ccr1^−/−^* kidneys late after infection (Figure 4), and from our competitive repopulation study, which showed that, despite the significant skewing toward *Ccr1^+/+^* neutrophils, *Ccr1^−/−^* neutrophils also trafficked from the blood into the kidney at day 9 post-infection (Figure 8). Thus, future studies should aim to determine which chemotactic factors, in addition to Ccr1, also mediate neutrophil trafficking from the blood into the kidney late after infection. Identification of such factors may reveal other novel mediators of immunopathology in the model and could provide further insight into the balance between effective host defense and immunopathology in invasive candidiasis.

In contrast to the mouse model of the infection where kidney is the primary target organ [5], kidney abscesses are rare in patients with bloodstream-derived invasive candidiasis with the exception of neonates [49]; hence, whether CCR1 mediates immunopathology in human invasive candidiasis remains to be elucidated. Therefore, future studies should examine whether CCR1 is expressed on human neutrophils in *Candida* kidney abscesses. In addition, because neutropenic patients with gastrointestinal tract-derived hepatosplenic candidiasis often develop worsening liver and splenic abscesses immediately after neutrophil recovery [7], the role of Ccr1 in mediating immunopathology in that setting should be investigated using a neutropenic mouse model of gastrointestinal tract-derived invasive candidiasis [50], in which it would be important to determine whether neutrophils that repopulate the bloodstream after recovery from neutropenia express Ccr1.

In conclusion, our data identify Ccr1 as a critical pathogenic factor in systemic infection caused by *Candida albicans* in a mouse model. Our study shows for the first time that neutrophil-mediated immunopathology may be detrimental in an invasive fungal infection and that chemokine receptors regulate such a process. Further studies will be needed to investigate the role of Ccr1 ligands and to further understand the factors that shape and modulate protective versus pathogenic roles of neutrophils in invasive candidiasis.

## Materials and Methods

### Ethics statement

The mouse studies were performed in strict accordance with the recommendations in the Guide for the Care and Use of Laboratory Animals of the National Institutes of Health under the auspices of a protocol approved by the Animal Care and Use Committee of the National Institute of Allergy and Infectious Diseases. Every effort was made to minimize animal suffering.

### Mouse model of invasive candidiasis

Female *Ccr1* ^+/+^ WT and *Ccr1* ^−/−^ C57BL/6 mice were obtained from Taconic Farms (Germantown, NY) and were used at 8–10 weeks of age. *Ccr1* ^−/−^ mice, generated as previously described [27], were backcrossed onto the C57BL/6 background for 10 generations and maintained under specific pathogen-free housing conditions. Mice were injected via the lateral tail vein with 1.25 or 2.5×10^5^ blastospores of *C. albicans* SC5314 (kind gift of Dr. John Bennett, NIAID, NIH). *Candida* was grown at 30°C in yeast extract, peptone, and dextrose medium containing penicillin and streptomycin (Mediatech Inc., Herndon, VA). Cells were centrifuged, washed in PBS, and counted using a hemocytometer.

### Fungal burden determination

Infected mice were euthanized at 1, 6 and 9 days post-infection to determine the fungal burden in the kidney, brain, spleen and liver. The organs were aseptically removed, weighed, homogenized in PBS, serially diluted, and plated in duplicate on yeast extract, peptone, and dextrose agar plates containing penicillin and streptomycin. CFUs were determined after 48 hrs of incubation at 37°C and results were expressed as CFUs/gram of tissue.

### Mouse histology and immunohistochemistry (IHC)

Mice were euthanized on day 9 post-infection and the kidney, brain, spleen and liver were removed, fixed with 10% formalin and embedded in paraffin wax. Tissue sections were processed for hematoxylin and eosin staining or Periodic-acid Schiff or were placed on poly-L-lysine coated glass slides, deparaffinized in xylene and rehydrated in a graded series of alcohol. After antigen retrieval for 20 min at 85°C, endogenous peroxidase was blocked by alcohol containing 0.3% hydrogen peroxide for 10 min at room temperature (RT). The slides were incubated with 5% BSA (Sigma-Aldrich, St. Louis, MO) for 20 min to block nonspecific protein binding and washed with Tris-buffered saline containing 0.05% Tween 20. Then, rat anti-mouse 7/4 (1∶10; Cedarlane Laboratories, Burlington, NC) was added in 1% BSA and slides were incubated overnight at RT. 1% BSA without primary antibody was used as negative control. The slides were washed twice with Tris-buffered saline containing 0.05% Tween 20 and detection of immunoreaction was achieved using the streptavidin-horse radish peroxidase system (Dako, Carpinteria, CA) containing streptavidin-peroxidase complex after a 30 min incubation at RT. Color was developed with 3,3′-diaminobenzidine (Dako) and hydrogen peroxide (Sigma), and slides were subsequently counterstained with hematoxylin, dehydrated, and mounted. At least three different sections of each organ per time-point were tested per experiment.

### Determination of serum blood urea nitrogen (BUN) and creatinine

Serum BUN and creatinine were measured as markers of renal function at day 9 post-infection in *Ccr1^+/+^* and *Ccr1* ^−/−^ mice. Blood was collected by cardiac puncture from each mouse at the time of sacrifice, and stored at −80°C until use. BUN and creatinine concentrations were determined at the NIH Clinical Chemistry Laboratory.

### Chemoattractant receptor, chemokine and adhesion molecule gene expression analysis

In the initial mRNA analysis of the chemokine system in *Ccr1^+/+^* mice, control uninfected mice and mice infected with 2.5×10^5^ CFUs of *Candida* were euthanized on day 1, 4, and 7 post-infection (n = 3 mice per time point) and mRNA was extracted from the kidney, brain, liver and spleen using Trizol (Invitrogen, Carlsbad, CA) and the RNeasy kit (Qiagen, Valencia, CA) according to the manufacturer's instructions. cDNA was generated using the Superscript III First-Strand Synthesis SuperMix kit with random hexamers (Invitrogen) and real-time quantitative PCR (qPCR) for chemoattractant receptors and ligands was performed by SYBR Green or Taqman detection (Applied Biosystems, Foster City, CA) with the 7900HT Fast Real-Time PCR System in a total reaction volume of 22 µl using 2 µl cDNA, 11 µl 2× SYBR Green or Taqman PCR Master mix, and 1.1 µl of forward primer/1.1 µl of reverse primer (SYBR Green) or 1.1 µl primer–probe mix (Taqman). All qPCR assays were performed in duplicate and the relative gene expression of each molecule compared to uninfected mice was determined after normalization with GAPDH transcript levels using the ΔΔCT method, displayed as either gene amplicons or mRNA fold change. The primers using SYBR Green detection were designed from GenBank sequences using software Primer3 (http://frodo.wi.mit.edu/primer3/) and purchased from Invitrogen (sequences shown in Table S1), whereas Taqman primers/probes were predesigned by Applied Biosystems.

To determine the relative gene expression of chemoattractant receptors, chemokines and adhesion molecules in *Ccr1^+/+^* versus *Ccr1* ^−/−^ mice, uninfected mice and mice infected with 1.25×10^5^ CFUs of *Candida* were euthanized on day 3, 6, and 9 post-infection (n = 3–5 mice per time point) and qPCR was performed as mentioned above in two independent experiments. Moreover, to determine the extent of kidney epithelial cell injury in *Ccr1^+/+^* versus *Ccr1* ^−/−^ mice, the relative expression of KIM-1 was determined at day 9 post-infection using the previously published *Kim-1* primer pairs: 5′-ATGAATCAGATTCAAGTCTTC-3′, 5′-TCTGGTTTGTGAGTCCATGTG-3′ [51].

### Chemokine quantification

Kidneys were homogenized into PBS with 0.5% Tween 20 (Bio-Rad, Hercules, CA) and a protease inhibitor cocktail (Roche, San Francisco, CA) and centrifuged at 13,000× rpm for 10 min at 4°C. The supernatants were aliquoted and frozen at −80°C until use. Mouse CCL3, CCL5, CCL8 and CCL9 were quantified using Duoset kits (R&D Systems, Minneapolis, MN). Mouse CCL6 was quantified by sandwich ELISA using a kit according to the manufacturer's instructions (Antigenix America, Huntington Station, NY), and mouse CCL7 was measured using the CCL7 Instant ELISA kit (BenderMed Systems, Vienna, Austria).

### Single cell suspension from blood, urine and organs

Mice were euthanized on day 3, 6, 9 and 12 post-infection (n = 3 mice/time-point). Urine was collected by applying external pressure over the urinary bladder and the volume obtained was recorded. Mice were anesthetized using ketamine/xylazine and 300 µl of blood were obtained via cardiac puncture in EDTA tubes. Then, the animals were perfused with normal saline and organs were harvested. Single cell suspensions were obtained using previously described methods [8]. In brief, anti-coagulated peripheral blood was treated with 1× PharmLyse buffer (BD Biosciences, San Jose, CA) according to the manufacturer's protocol to remove red blood cells. Then, leukocytes were washed three times with HBSS+2 mM EDTA+1% BSA, suspended in PBS, and passed though a 40-µm filter (BD Biosciences). Urine leukocytes were washed three times with RPMI+10% FBS, suspended in PBS, and passed through a 40-µm filter. Brains were collected in 7 ml of FACS buffer and homogenized using the back of a 6-cc syringe on ice in a tissue culture plate. The suspension was brought to 10 ml by adding 3 ml of 90% Percoll (GE Healthcare, Piscataway, NJ) in PBS. After thorough mixing, the solution was underlaid with 1 ml of 70% Percoll (in PBS), and was centrifuged at 2,450 rpm for 30 min at 4°C. The leukocytes at the interphase were isolated, washed three times in FACS buffer, suspended in PBS, and passed through a 40-µm filter. Spleens were finely minced and digested at 37°C in digestion solution (RPMI 1640 with 20 mM HEPES without serum) containing liberase TL (Roche Diagnostics, Chicago, IL) and grade II DNAse I (Roche) for 20 min with shaking. Digested tissue was passed through a 100-µm filter and washed, and the remaining red cells were lysed with ACK lysing buffer (Lonza, Walkersville, MD). Then, cells were passed through a 70-µm filter and washed three times with FACS buffer. Finally, splenocytes were suspended in PBS and passed through a 40-µm filter. To obtain kidney and liver single cell suspensions, the organs were finely minced and digested at 37°C in digestion solution containing liberase TL and grade II DNAse I for 20 min with shaking. Digested tissue was passed through a 70-µm filter, washed, and the remaining red cells were lysed with ACK lysing buffer for 30 seconds. Then, the cells were passed through a 40-µm filter, washed, and suspended in 40% Percoll. The suspension was overlaid on 70% Percoll, and centrifuged at 2,000 rpm for 30 min at RT. The leukocytes at the interphase were isolated, washed three times in FACS buffer, suspended in PBS, and passed though a 40-µm filter.

### Flow cytometry

The blood, urine, kidney, brain, spleen and liver single cell suspensions were stained with a Live/Dead fluorescent dye (Invitrogen, Carlsbad, CA) for 10 min (1∶500) and incubated with rat anti-mouse CD16/32 (2.4G2; BD Biosciences) for 10 min (1∶100) at 4°C to block Fc receptors. Then, cells were incubated at 4°C for 30 min with the following anti-mouse antibodies: efluor450 CD45 (Ly-5; eBioscience, San Diego, CA); FITC-conjugated Ly6c (AL-21), CD3 (17A2; BD Biosciences) and MHC II (M5/114.15.2; eBioscience); PE-conjugated CD45 (Ly-5), Ly6G (1A8), NK1.1 (PK136; BD Biosciences); PE-Cy7-conjugated F4/80 (BM8) or CD8 (53-6.7; eBioscience); allophycocyanin-conjugated CD45 (Ly-5), CD11c (HL3) (BD Biosciences) or CD19 (1D3; eBioscience); allophycocyanin-Cy7-conjugated CD11b (M1/70; BD Biosciences) or APC-eFluor 780-conjugated CD4 (RM4-5; eBioscience). After three washes with FACS buffer, cells were fixed with 2% paraformaldehyde. FACS was performed on an LSRII (BD Biosciences) and data were analyzed using FlowJo (version 8.8.4; Treestar, Ashland, OR). Cell numbers were quantified using PE-conjugated fluorescent counting beads (Spherotech, Lake Forest, IL).

### FACS analysis of neutrophils for annexin V and 7-AAD expression

To determine whether Ccr1 deficiency influenced neutrophil survival post-infection, we performed FACS analysis on kidney and spleen neutrophils from *Ccr1* ^+/+^ and *Ccr1* ^−/−^ mice on days 6 and 9 post-infection. Cells were initially incubated for 10 min with rat anti-mouse CD16/32 and then stained for 30 min with the combination of PE-conjugated Ly6G, allophycocyanin-conjugated CD45 and allophycocyanin-Cy7-conjugated CD11b (BD Biosciences). Then, the cells were washed twice with PBS, suspended in 1× Annexin V binding buffer (BD Biosciences), and incubated for 15 min with 5 µl each of FITC-conjugated Annexin V and 7-AAD according to the manufacturer's instructions (BD Biosciences). The cells were washed and FACS was performed on an LSRII.

### FACS analysis of neutrophils for Ccr1 expression

To determine the expression of Ccr1 on neutrophils and other leukocytes after infection, we performed FACS analysis on kidney and blood cells on days 3, 6 and 9 post-infection. Cells were initially incubated for 15 min with rat anti-mouse CD16/32 and then stained for 30 min with efluor450-conjugated CD45 or CD3, FITC-conjugated Ly6c or Ly6G, PE-Cy7-conjugated F4/80, allophycocyanin-conjugated CD19, Ly6G, CD45 or CD11c, allophycocyanin-Cy7-conjugated CD11b, and PE-conjugated rat monoclonal anti-mouse Ccr1 (FAB5986P, clone 643854; R&D Systems) or PE-conjugated isotype control (rat IgG2B; IC013P, clone 141945; R&D Systems) according to the manufacturer's protocol. The cells were washed and FACS was performed on an LSRII. The specificity of the monoclonal Ccr1 antibody was confirmed by demonstration of binding on Ccr1-transfected, but not untransfected control, HEK293 cells (data not shown).

### Magnetic sorting of mouse kidney neutrophils

To sort kidney neutrophils the AutoMACS automatic magnetic cell-sorting system was used. Kidney single cell suspensions from *Ccr1^+/+^* mice euthanized on days 3, 6 and 9 post-infection were prepared as described above, and the kidney cells were incubated with the anti-Ly6G MicroBead kit (Miltenyi Biotec, Auburn, CA) according to the manufacturer's instructions. Ly6G^+^ cell purification was conducted on an AutoMACS separator (Miltenyi Biotec) consistently achieving an enriched population of >90% Ly6c^int^Ly6G^+^CD11b^+^ neutrophils with >95% viability (data not shown).

### Gene expression of Ccr1 and its ligands on kidney neutrophils after infection

mRNA was extracted from the MACS-purified kidney neutrophils of *Ccr1^+/+^* mice at days 3, 6 and 9 post-infection (n = 3–5 per time-point) using Trizol per the manufacturer's protocol. cDNA was generated, and qPCR was performed for Ccr1, Ccl3, Ccl5, Ccl6, Ccl7, Ccl8 and Ccl9 as described above. Relative gene expression was determined after normalization with GAPDH transcript levels using the ΔΔCT method and displayed as gene amplicons.

### Chemotaxis assay

Chemotaxis was measured using a Boyden Chamber assay. Briefly, MACS-purified mouse neutrophils from 12 pooled kidneys of 6 *Ccr1^+/+^* mice at day 9 post-infection (50 µl, 1×10^6^ cells/ml) were added to the upper chamber and recombinant mouse CCL3, CCL5, CCL7, CCL8 (Peprotech Inc., Rocky Hill, NJ), CCL6 or CCL9 (R&D Systems) (concentration range: 0–500 nM) was added to the lower chamber separated by a membrane containing 3 µm diameter pores (Neuro Probe Inc., Gaithersburg, MD). Following a 1 hr incubation in a 37°C, 5% CO_2_ incubator, chemotaxis was assessed in triplicate by counting the number of migrated cells in three random fields per well.

### *In vivo* neutrophil adoptive transfer

Marrow cavities of the tibias and femurs of *Candida*-infected *Ccr1* ^+/+^ and *Ccr1* ^−/−^ mice at day 9 post-infection were flushed with RPMI+10% FBS+2 mM EDTA. Following hypotonic red blood cell lysis with 0.2% and 1.6% NaCl, mature neutrophils were isolated by gradient centrifugation over Histopaque 1119 (density, 1.119 g/mL) and Histopaque 1077 (density, 1.077 g/mL) according to the manufacturer's instructions at 2000 rpm for 30 min at 25°C. Neutrophils recovered at the interface of the Histopaque 1119 and Histopaque 1077 layers were 80–90% pure and >95% viable as determined by FACS (data not shown). Neutrophils were washed twice with RPMI+10% FBS+penicillin/streptomycin and were resuspended at 5×10^6^ cells/ml in PBS. *Ccr1^+/+^* and *Ccr1^−/−^* neutrophils were then incubated with CMFDA Cell Tracker Orange (Invitrogen) and CMTMR Cell Tracker Green (Invitrogen), respectively, in a water bath at 37°C for 10 min (final concentration, 5 µM). Cells were washed with cold RPMI+10% FBS+penicillin/streptomycin three times, resuspended in sterile PBS at a final concentration of 40×10^6^ cells/ml, and mixed at a 1∶1 ratio. A total of 8×10^6^ neutrophils (200 µl) were injected intravenously per animal in 16 *Ccr1^+/+^ Candida*-infected mice on day 9 post-infection and 4 hours later, the mice were euthanized, their kidneys were harvested and single cell suspensions were prepared as described above. After live/dead staining and Fc blockade, leukocytes were stained with efluor450-conjugated CD45, allophycocyanin-conjugated Ly6G and allophycocyanin-Cy7 conjugated CD11b. Neutrophil migration was calculated based on the ratio detected in the kidney compared with the initial input ratio of labeled cells at the time of cell transfer (*Ccr1^+/+^*:*Ccr1* ^−/−^ cells) after gating on CD45^+^Ly6G^+^CD11b^+^ cells.

### Beta-glucuronidase assay

The degranulation potential of *Candida*-infected kidney neutrophils was assessed using the beta-glucuronidase assay as previously described [52]. Kidney neutrophils of *Ccr1^+/+^* mice were magnetically sorted as mentioned above at days 3 (Ccr1^lo^) and 9 (Ccr1^high^) post-infection. Then, 5×10^4^ neutrophils were lysed using 0.2% Triton-X-100 and incubated for 30 minutes in black 96-well plates with 4-methylumbelliferyl-β-D-glucuronide (MUB-glucuronide; Sigma) in a 37°C water bath. The reaction was terminated by adding ice-cold glycine (50 mM) and ammonium hydroxide (200 mM) buffer (pH, 10.5). The neutrophil β-glucuronidase activity was then determined by measuring the chromogenic substrate β-methylumbelliferone (MUB; Sigma) using a fluorescent plate reader with excitation wavelength of 360 nm and emission wavelength of 455 nm. Two independent experiments were performed using a total of 6–10 mice per time-point.

### Neutrophil oxidative burst

The oxidative burst of *Candida*-infected kidney neutrophils was assessed using the DHR assay as previously described [53]. Kidney neutrophils of *Ccr1^+/+^* mice were magnetically sorted as mentioned above at days 3 (Ccr1^lo^) and 9 (Ccr1^high^) post-infection. Then, 2.5×10^5^ neutrophils were suspended in sterile HBSS buffer (i.e., HBSS without calcium/magnesium/phenol red, plus 0.5 g of BSA, plus 1 mM of EDTA) and 2 µl of a 25 mM DHR solution (Invitrogen) was added. After a 5-minute incubation at 37°C, PMA (Sigma) was then added (final concentration, 400 ng/ml) and the neutrophils were incubated in a 37°C shaking water bath for 15 minutes. Neutrophils not stimulated with PMA were also tested to determine the “resting” levels of rhodamine 123 fluorescence. The mean fluorescence intensity was then analyzed using flow cytometry in the FL-1 channel as a measure of the conversion of the non-fluorescent reactive oxygen species indicator DHR to the oxidized cationic rhodamine 123, which exhibits green fluorescence. Two independent experiments were performed using a total of 6–10 mice per time-point.

### Neutrophil phagocytosis and killing assays

Kidney neutrophils of *Ccr1^+/+^* mice were magnetically sorted as mentioned above at days 3 (Ccr1^lo^) and 9 (Ccr1^high^) post-infection. For the phagocytosis assays, 5×10^5^ neutrophils were incubated for 30 min at 37°C with 5×10^5^ yeast cells of opsonized GFP-*Candida albicans* strain SC5314 (kind gift from Dr. Robert Wheeler, University of Maine) at a neutrophil∶*Candida* ratio of 1∶1. The percent of *Candida* phagocytosis was then determined using flow cytometry by gating on the FITC-positive neutrophils. For the killing assays, 5×10^5^ neutrophils were plated on 96-well flat bottom plates for 30 minutes to create a monolayer and then 10^5^ yeast cells of opsonized *Candida albicans* strain SC5314 were added at a neutrophil∶*Candida* ratio of 5∶1. Following 30 minutes of phagocytosis at 37°C, the non-phagocytosed *Candida* cells were washed out of the wells. The neutrophil anti-*Candida* killing capacity was then determined by plating the number of viable *Candida* cells after an additional 90-minute incubation at 37°C as previously described [54]. Two independent experiments were performed using a total of 7–10 mice per time-point for the phagocytosis assays and a total of 8–10 mice per time-point for the killing assays.

### Statistical analysis

The data were analyzed using the two-tailed unpaired *t*-test or the Mann-Whitney test where appropriate with Prism 5.0 software (GraphPad Software, San Diego, CA) and are presented as the mean ± SEM. The cutoff for statistical significance was defined as *P*<0.05.

## Supporting Information

Figure S1 **Effect of Ccr1 deficiency on organ immunopathology and** ***Candida*** **morphology in the kidney in a mouse model of invasive candidiasis.** (A) Ccr1 deficiency does not affect the morphology of *Candida* infiltration in infected kidneys. Representative Periodic acid-Schiff staining of the renal pelvis of *Ccr1^+/+^* and *Ccr1^−/−^* mice at day 9 post-infection showing *Candida* hyphal formation (cross section; magnification, ×600) (B) Ccr1 deficiency does not affect organ weight in the spleen, liver or brain post-infection. (C) Hematoxylin and Eosin staining of uninfected mouse kidney (cross section; magnification, ×20) for comparison. (D) Hematoxylin and Eosin staining of the renal cortex from *Ccr1^+/+^* and *Ccr1^−/−^* mice at day 9 post-infection showing the greater tubular cast formation and tissue damage in *Ccr1^+/+^* kidneys. The images represent higher magnifications (×600) of the bottom row images of Figure 3C. (E) Hematoxylin and Eosin staining of uninfected kidney cortex (cross section; magnification, ×400) for comparison. (F) Ccr1 deficiency does not affect tissue damage in the spleen, liver or brain post-infection. Shown is Hematoxylin and Eosin staining in these organs at day 9 post-infection (magnification, ×400).
(TIF)

Figure S2 **Ccr1 deficiency does not significantly affect the accumulation of neutrophils in spleen, liver or brain post-infection in a mouse model of invasive candidiasis.** (A) spleen, (B) liver, (C) brain. Data are from two to three independent experiments using six to nine *Ccr1^+/+^* and six to nine *Ccr1^−/−^* mice per time-point.
(TIF)

Figure S3 **Ccr1 deficiency only affects accumulation of neutrophils in the kidney in a mouse model of invasive candidiasis.** Accumulation is shown for (A) inflammatory monocytes, (B) NK cells, (C) macrophages, (D) dendritic cells, (E) CD4^+^ T cells, (F) CD8^+^ T cells, and (G) B cells in the kidney post-*Candida* infection. Data are from two to four independent experiments using six to twelve *Ccr1^+/+^* and six to twelve *Ccr1^−/−^* mice per time-point.
(TIF)

Figure S4 **Ccr1 deficiency does not affect survival of neutrophils in the spleen after** ***Candida*** **infection.** Percent of (A) live annexin V*^−^* 7-AAD*^−^*, (B) apoptotic annexin V*^+^* 7-AAD*^−^*, and (C) dead annexin V*^+^* 7-AAD*^+^* splenic neutrophils is similar in *Ccr1^+/+^* and *Ccr1^−/−^* mice at days 6 and 9 post-infection. Data are shown from one of two independent experiments with similar pattern of results using a total of seven *Ccr1^+/+^* and seven *Ccr1^−/−^* mice per time-point.
(TIF)

Figure S5 **Ccr1 expression on leukocyte subsets in the kidney in a mouse model of invasive candidiasis.** (A) monocytes, (B) macrophages, (C) dendritic cells, (D) T cells and (E) B cells. Data are from day 6 post-infection and are representative FACS histograms from two independent experiments using four to six *Ccr1^+/+^* and four to six *Ccr1^−/−^* mice per time-point.
(TIF)

Figure S6 **Ccr1 deficiency does not affect expression of Ccr1 ligands in the spleen in a mouse model of invasive candidiasis.** Data are from one experiment using four *Ccr1^+/+^* and four *Ccr1^−/−^* mice per time-point.
(TIF)

Figure S7 **Ccr1 deficiency does not decrease the expression of other neutrophil-targeted chemotactic factors or adhesion molecules in** ***Candida*** **-infected kidneys.** Relative mRNA expression is shown for the indicated factors in *Ccr1^+/+^* and *Ccr1^−/−^* kidneys at days 3 (A–C) and 6 (D–F) after *Candida* infection. Data are from two independent experiments using seven *Ccr1^+/+^* and seven *Ccr1^−/−^* mice per time-point. ^*^ *P* = 0.02.
(TIF)

Figure S8 **Ccr1 expression on kidney neutrophils does not significantly affect their immunopathogenic potential or anti-** ***Candida*** **effector function.** Sorted Ccr1^lo^ (day 3) and Ccr1^high^ (day 9) kidney neutrophils do not differ in their capacity for degranulation (A), oxidative burst (B), *Candida* phagocytosis (C) or anti-*Candida* killing (D). Data are from two independent experiments using 6–10 *Ccr1^+/+^* mice per time-point for degranulation and oxidative burst assays, 7–10 *Ccr1^+/+^* mice per time-point for phagocytosis assays, and 8–10 *Ccr1^+/+^* mice per time-point for killing assays.
(TIF)

Table S1 **Sequences of the primers used for qPCR with SYBR Green in the present study.**
(PDF)

## References

[1] Zaoutis TE, Argon J, Chu J, Berlin JA, Walsh TJ, et al (2005) The epidemiology and attributable outcomes of candidemia in adults and children hospitalized in the United States: a propensity analysis. Clin Infect Dis 41: 1232–1239. 16206095 10.1086/496922

[2] Miller LG, Hajjeh RA, Edwards JE Jr (2001) Estimating the cost of nosocomial candidemia in the united states. Clin Infect Dis 32: 1110. 10.1086/319613 11264044

[3] Wilson LS, Reyes CM, Stolpman M, Speckman J, Allen K, et al (2002) The direct cost and incidence of systemic fungal infections. Value Health 5: 26–34. 11873380 10.1046/j.1524-4733.2002.51108.x

[4] Pappas PG (2006) Invasive candidiasis. Infect Dis Clin North Am 20: 485–506. 16984866 10.1016/j.idc.2006.07.004

[5] Spellberg B, Ibrahim AS, Edwards JE Jr, Filler SG (2005) Mice with disseminated candidiasis die of progressive sepsis. J Infect Dis 192: 336–343. 15962230 10.1086/430952

[6] Szabo EK, MacCallum DM (2011) The contribution of mouse models to our understanding of systemic candidiasis. FEMS Microbiol Lett 320: 1–8. 21395661 10.1111/j.1574-6968.2011.02262.x

[7] Legrand F, Lecuit M, Dupont B, Bellaton E, Huerre M, et al (2008) Adjuvant corticosteroid therapy for chronic disseminated candidiasis. Clin Infect Dis 46: 696–702. 18230039 10.1086/527390

[8] Lionakis MS, Lim JK, Lee CC, Murphy PM (2011) Organ-specific innate immune responses in a mouse model of invasive candidiasis. J Innate Immun 3: 180–199. 21063074 10.1159/000321157 PMC3072204

[9] Fulurija A, Ashman RB, Papadimitriou JM (1996) Neutrophil depletion increases susceptibility to systemic and vaginal candidiasis in mice, and reveals differences between brain and kidney in mechanisms of host resistance. Microbiology 142: 3487–3496. 9004511 10.1099/13500872-142-12-3487

[10] Qian Q, Jutila MA, Van Rooijen N, Cutler JE (1994) Elimination of mouse splenic macrophages correlates with increased susceptibility to experimental disseminated candidiasis. J Immunol 152: 5000–5008. 8176217

[11] Mahanty S, Greenfield RA, Joyce WA, Kincade PW (1988) Inoculation candidiasis in a murine model of severe combined immunodeficiency syndrome. Infect Immun 56: 3162–3166. 3182076 10.1128/iai.56.12.3162-3166.1988 PMC259718

[12] Winkelstein JA, Marino MC, Johnston RB Jr, Boyle J, Curnutte J, et al (2000) Chronic granulomatous disease. Report on a national registry of 368 patients. Medicine (Baltimore) 79: 155–169. 10844935 10.1097/00005792-200005000-00003

[13] Vonk AG, Netea MG, van Krieken JH, Iwakura Y, van der Meer JW, et al (2006) Endogenous interleukin (IL)-1 alpha and IL-1 beta are crucial for host defense against disseminated candidiasis. J Infect Dis 193: 1419–1426. 16619190 10.1086/503363

[14] Stuyt RJ, Netea MG, Verschueren I, Fantuzzi G, Dinarello CA, et al (2002) Role of interleukin-18 in host defense against disseminated *Candida albicans* infection. Infect Immun 70: 3284–3286. 12011026 10.1128/IAI.70.6.3284-3286.2002 PMC127971

[15] Netea MG, van Tits LJ, Curfs JH, Amiot F, Meis JF, et al (1999) Increased susceptibility of TNF-alpha lymphotoxin-alpha double knockout mice to systemic candidiasis through impaired recruitment of neutrophils and phagocytosis of *Candida albicans* . J Immunol 163: 1498–1505. 10415052

[16] van Enckevort FH, Netea MG, Hermus AR, Sweep CG, Meis JF, et al (1999) Increased susceptibility to systemic candidiasis in interleukin-6 deficient mice. Med Mycol 37: 419–426. 10647123 10.1046/j.1365-280x.1999.00247.x

[17] Mencacci A, Cenci E, Del Sero G, Fé d'Ostiani C, Mosci P, et al (1998) IL-10 is required for development of protective Th1 responses in IL-12-deficient mice upon *Candida albicans* infection. J Immunol 161: 6228–6237. 9834110

[18] Lavigne LM, Schopf LR, Chung CL, Maylor R, Sypek JP (1998) The role of recombinant murine IL-12 and IFN-gamma in the pathogenesis of a murine systemic *Candida albicans* infection. J Immunol 160: 284–292. 9551982

[19] Vazquez-Torres A, Jones-Carson J, Wagner RD, Warner T, Balish E (1999) Early resistance of interleukin-10 knockout mice to acute systemic candidiasis. Infect Immun 67: 670–674. 9916075 10.1128/iai.67.2.670-674.1999 PMC96371

[20] Balish E, Warner TF, Nicholas PJ, Paulling EE, Westwater C, et al (2005) Susceptibility of germfree phagocyte oxidase- and nitric oxide synthase 2-deficient mice, defective in the production of reactive metabolites of both oxygen and nitrogen, to mucosal and systemic candidiasis of endogenous origin. Infect Immun 73: 1313–1320. 15731028 10.1128/IAI.73.3.1313-1320.2005 PMC1064974

[21] Huang W, Na L, Fidel PL, Schwarzenberger P (2004) Requirement of interleukin-17A for systemic anti-*Candida albicans* host defense in mice. J Infect Dis 190: 624–631. 15243941 10.1086/422329

[22] Murphy PM, Baggiolini M, Charo IF, Hébert CA, Horuk R, et al (2000) International union of pharmacology. XXII. Nomenclature for chemokine receptors. Pharmacol Rev 52: 145–176. 10699158

[23] Tsou CL, Peters W, Si Y, Slaymaker S, Slaymaker S, et al (2007) Critical roles for CCR2 and MCP-3 in monocyte mobilization from bone marrow and recruitment to inflammatory sites. J Clin Invest 117: 902–909. 17364026 10.1172/JCI29919 PMC1810572

[24] Ichimura T, Bonventre JV, Bailly V, Wei H, Hession CA, et al (1998) Kidney injury molecule-1 (KIM-1), a putative epithelial cell adhesion molecule containing a novel immunoglobulin domain, is up-regulated in renal cells after injury. J Biol Chem 273: 4135–4142. 9461608 10.1074/jbc.273.7.4135

[25] Vaidya VS, Ozer JS, Dieterle F, Collings FB, Ramirez V, et al (2010) Kidney injury molecule-1 outperforms traditional biomarkers of kidney injury in preclinical biomarker qualification studies. Nat Biotechnol 28: 478–485. 20458318 10.1038/nbt.1623 PMC2885849

[26] Karlmark KR, Zimmermann HW, Roderburg C, Gassler N, Wasmuth HE, et al (2010) The fractalkine receptor CX3CR1 protects against liver fibrosis by controlling differentiation and survival of infiltrating hepatic monocytes. Hepatology 52: 1769–1782. 21038415 10.1002/hep.23894

[27] Gao JL, Wynn TA, Chang Y, Lee EJ, Broxmeyer HE, et al (1997) Impaired host defense, hematopoiesis, granulomatous inflammation and type 1-type 2 cytokine balance in mice lacking CC chemokine receptor 1. J Exp Med 185: 1959–1968. 9166425 10.1084/jem.185.11.1959 PMC2196337

[28] Gerard C, Frossard JL, Bhatia M, Saluja A, Gerard NP, et al (1997) Targeted disruption of the beta-chemokine receptor CCR1 protects against pancreatitis-associated lung injury. J Clin Invest 100: 2022–2027. 9329966 10.1172/JCI119734 PMC508392

[29] He M, Horuk R, Moochhala SM, Bhatia M (2007) Treatment with BX471, a CC chemokine receptor 1 antagonist, attenuates systemic inflammatory response during sepsis. Am J Physiol Gastrointest Liver Physiol 292: 1173–1180. 10.1152/ajpgi.00420.2006 17234893

[30] Zhang S, Youn BS, Gao JL, Murphy PM, Kwon BS (1999) Differential effects of leukotactin-1 and macrophage inflammatory protein-1 alpha on neutrophils mediated by CCR1. J Immunol 162: 4938–4942. 10202040

[31] Schuit KE (1979) Phagocytosis and intracellular killing of pathogenic yeasts by human monocytes and neutrophils. Infect Immun 24: 932–938. 381207 10.1128/iai.24.3.932-938.1979 PMC414397

[32] Uzun O, Ascioglu S, Anaissie EJ, Rex JH (2001) Risk factors and predictors of outcome in patients with cancer and breakthrough candidemia. Clin Infect Dis 32: 1713–1717. 11360213 10.1086/320757

[33] Romani L, Mencacci A, Cenci E, Del Sero G, Bistoni F, et al (1997) An immunoregulatory role for neutrophils in CD4+ T helper subset selection in mice with candidiasis. J Immunol 158: 2356–2362. 9036985

[34] Miceli MH, Maertens J, Buvé K, Grazziutti M, Woods G, et al (2007) Immune reconstitution inflammatory syndrome in cancer patients with pulmonary aspergillosis recovering from neutropenia: Proof of principle, description, and clinical and research implications. Cancer 110: 112–120. 17525971 10.1002/cncr.22738

[35] Khan IA, Murphy PM, Casciotti L, Schwartzman JD, Collins J, et al (2001) Mice lacking the chemokine receptor CCR1 show increased susceptibility to *Toxoplasma gondii* infection. J Immunol 166: 1930–1937. 11160241 10.4049/jimmunol.166.3.1930

[36] Domachowske JB, Bonville CA, Gao JL, Murphy PM, Easton AJ, et al (2000) The chemokine macrophage-inflammatory protein-1 alpha and its receptor CCR1 control pulmonary inflammation and antiviral host defense in paramyxovirus infection. J Immunol 165: 2677–2682. 10946298 10.4049/jimmunol.165.5.2677

[37] Furuichi K, Gao JL, Horuk R, Wada T, Kaneko S, et al (2008) Chemokine receptor CCR1 regulates inflammatory cell infiltration after renal ischemia-reperfusion injury. J Immunol 181: 8670–8676. 19050287 10.4049/jimmunol.181.12.8670 PMC2633769

[38] Vielhauer V, Berning E, Eis V, Kretzler M, Segerer S, et al (2004) CCR1 blockade reduces interstitial inflammation and fibrosis in mice with glomerulosclerosis and nephrotic syndrome. Kidney Int 66: 2264–2278. 15569315 10.1111/j.1523-1755.2004.66038.x

[39] Ninichuk V, Gross O, Reichel C, Khandoga A, Pawar RD, et al (2005) Delayed chemokine receptor 1 blockade prolongs survival in collagen 4A3-deficient mice with Alport disease. J Am Soc Nephrol 16: 977–985. 15716328 10.1681/ASN.2004100871

[40] Eis V, Luckow B, Vielhauer V, Siveke JT, Linde Y, et al (2004) Chemokine receptor CCR1 but not CCR5 mediates leukocyte recruitment and subsequent renal fibrosis after unilateral ureteral obstruction. J Am Soc Nephrol 15: 337–347. 14747380 10.1097/01.asn.0000111246.87175.32

[41] Anders HJ, Vielhauer V, Frink M, Linde Y, Cohen CD, et al (2002) A chemokine receptor CCR-1 antagonist reduces renal fibrosis after unilateral ureter ligation. J Clin Invest 109: 251–259. 11805137 10.1172/JCI14040 PMC150841

[42] Topham PS, Csizmadia V, Soler D, Hines D, Gerard CJ, et al (1999) Lack of chemokine receptor CCR1 enhances Th1 responses and glomerular injury during nephrotoxic nephritis. J Clin Invest 104: 1549–1557. 10587518 10.1172/JCI7707 PMC409862

[43] Bonecchi R, Polentarutti N, Luini W, Borsatti A, Bernasconi S, et al (1999) Up-regulation of CCR1 and CCR3 and induction of chemotaxis to CC chemokines by IFN-gamma in human neutrophils. J Immunol 162: 474–479. 9886422

[44] Cheng SS, Lai JJ, Lukacs NW, Kunkel SL (2001) Granulocyte-macrophage colony stimulating factor up-regulates CCR1 in human neutrophils. J Immunol 166: 1178–1184. 11145699 10.4049/jimmunol.166.2.1178

[45] van de Veerdonk FL, Kullberg BJ, van der Meer JW, Gow NA, Netea MG (2008) Host-microbe interactions: innate pattern recognition of fungal pathogens. Curr Opin Microbiol 11: 305–312. 18602019 10.1016/j.mib.2008.06.002

[46] Wheeler RT, Kombe D, Agarwala SD, Fink GR (2008) Dynamic, morphotype-specific *Candida albicans* beta-glucan exposure during infection and drug treatment. PLoS Pathog 4: e1000227. 19057660 10.1371/journal.ppat.1000227 PMC2587227

[47] Chou RC, Kim ND, Sadik CD, Seung E, Lan Y, et al (2010) Lipid-cytokine-chemokine cascade drives neutrophil recruitment in a murine model of inflammatory arthritis. Immunity 33: 266–278. 20727790 10.1016/j.immuni.2010.07.018 PMC3155777

[48] Balish E, Wagner RD, Vazquez-Torres A, Jones-Carson J, Pierson C, et al (1999) Mucosal and systemic candidiasis in IL-8Rh−/− BALB/c mice. J Leukoc Biol 66: 144–150. 10411002 10.1002/jlb.66.1.144

[49] Bryant K, Maxfield C, Rabalais G (1999) Renal candidiasis in neonates with candiduria. Pediatr Infect Dis J 18: 959–963. 10571429 10.1097/00006454-199911000-00004

[50] Koh AY, Köhler JR, Coggshall KT, Van Rooijen N, Pier GB (2008) Mucosal damage and neutropenia are required for Candida albicans dissemination. PLoS Pathog 4: e35. 18282097 10.1371/journal.ppat.0040035 PMC2242836

[51] Ichimura T, Asseldonk EJ, Humphreys BD, Gunaratnam L, Duffield JS, et al (2008) Kidney injury molecule-1 is a phosphatidylserine receptor that confers a phagocytic phenotype on epithelial cells. J Clin Invest 118: 1657–1668. 18414680 10.1172/JCI34487 PMC2293335

[52] Kolotila MP, Diamond RD (1988) Stimulation of neutrophil actin polymerization and degranulation by opsonized and unopsonized *Candida albicans* hyphae and zymosan. Infect Immun 56: 2016–2022. 3294183 10.1128/iai.56.8.2016-2022.1988 PMC259517

[53] Jirapongsananuruk O, Malech HL, Kuhns DB, Niemela JE, Brown MR, et al (2003) Diagnostic paradigm for evaluation of male patients with chronic granulomatous disease, based on the dihydrorhodamine 123 assay. J Allergy Clin Immunol 111: 374–379. 12589359 10.1067/mai.2003.58

[54] Vonk AG, Netea MG, Kullberg BJ (2012) Phagocytosis and intracellular killing of *Candida albicans* by murine polymorphonuclear neutrophils. Methods Mol Biol 845: 277–287. 22328381 10.1007/978-1-61779-539-8_18

