# Supplementary figures and images for: Chemokine Receptor Ccr1 Drives Neutrophil-Mediated Kidney Immunopathology and Mortality in Invasive Candidiasis
Source: PLoS Pathog. 2012 Aug 16;8(8):e1002865. doi: 10.1371/journal.ppat.1002865 (PMC3420964; doi:10.1371/journal.ppat.1002865)

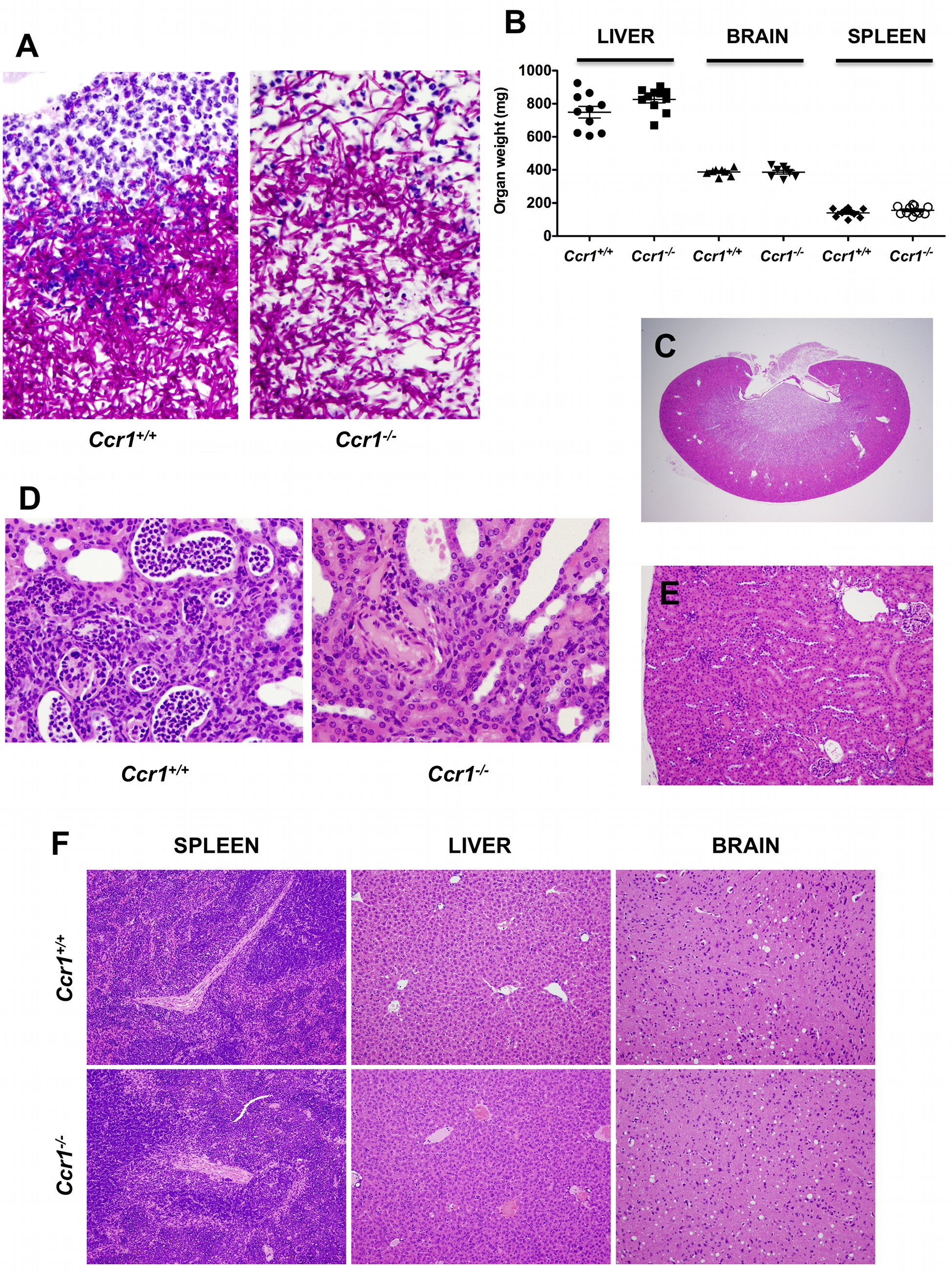

Supplement: Figure S1 — Effect of Ccr1 deficiency on organ immunopathology and Candida morphology in the kidney in a mouse model of invasive candidiasis. (A) Ccr1 deficiency does not affect the morphology of Candida infiltration in infected kidneys. Representative Periodic acid-Schiff staining of the renal pelvis of Ccr1+/+ and Ccr1−/− mice at day 9 post-infection showing Candida hyphal formation (cross section; magnification, ×600) (B) Ccr1 deficiency does not affect organ weight in the spleen, liver or brain post-infection. (C) Hematoxylin and Eosin staining of uninfected mouse kidney (cross section; magnification, ×20) for comparison. (D) Hematoxylin and Eosin staining of the renal cortex from Ccr1+/+ and Ccr1−/− mice at day 9 post-infection showing the greater tubular cast formation and tissue damage in Ccr1+/+ kidneys. The images represent higher magnifications (×600) of the bottom row images of Figure 3C. (E) Hematoxylin and Eosin staining of uninfected kidney cortex (cross section; magnification, ×400) for comparison. (F) Ccr1 deficiency does not affect tissue damage in the spleen, liver or brain post-infection. Shown is Hematoxylin and Eosin staining in these organs at day 9 post-infection (magnification, ×400). (TIF) [file ppat.1002865.s001.tif]

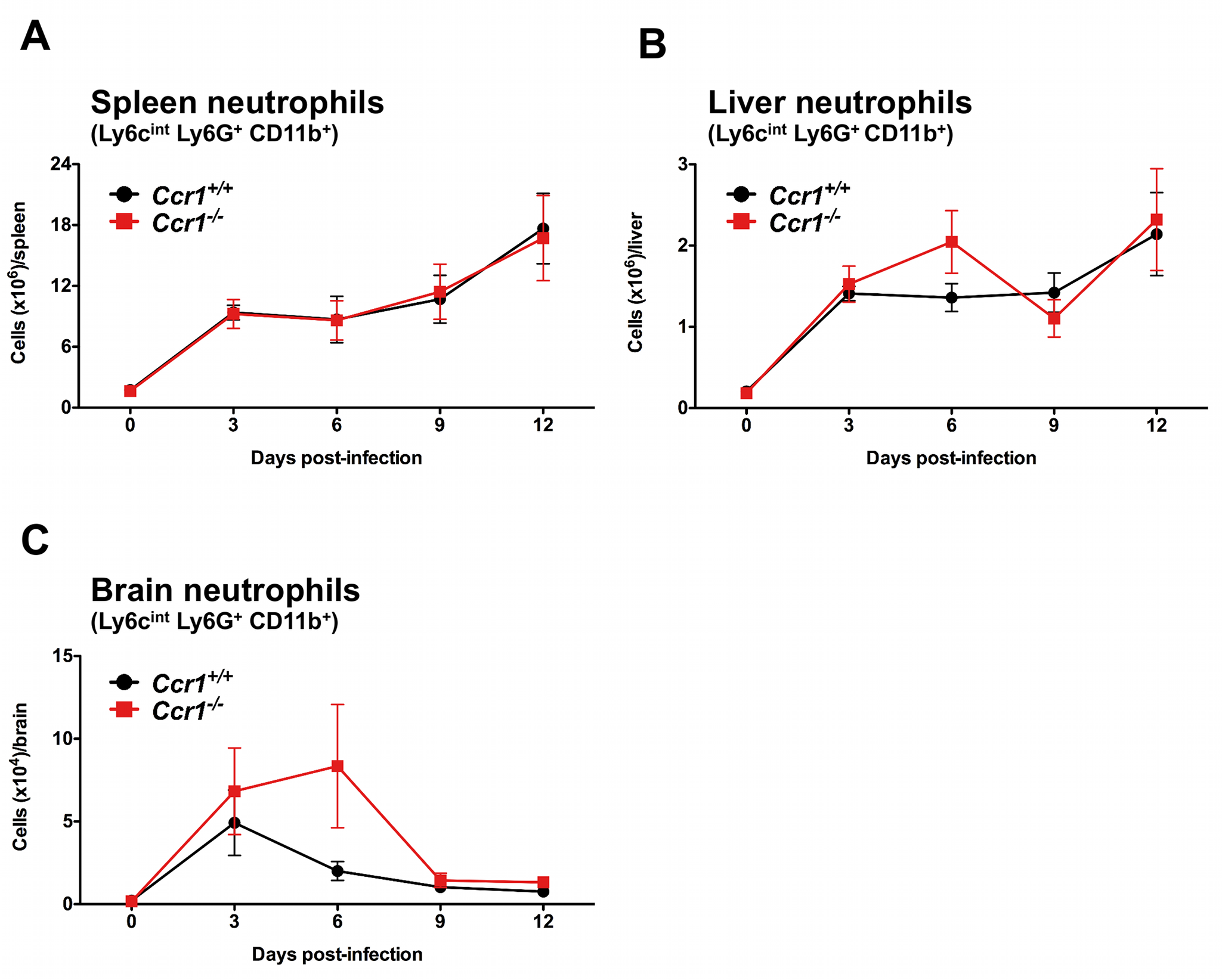

Supplement: Figure S2 — Ccr1 deficiency does not significantly affect the accumulation of neutrophils in spleen, liver or brain post-infection in a mouse model of invasive candidiasis. (A) spleen, (B) liver, (C) brain. Data are from two to three independent experiments using six to nine Ccr1+/+ and six to nine Ccr1−/− mice per time-point. (TIF) [file ppat.1002865.s002.tif]

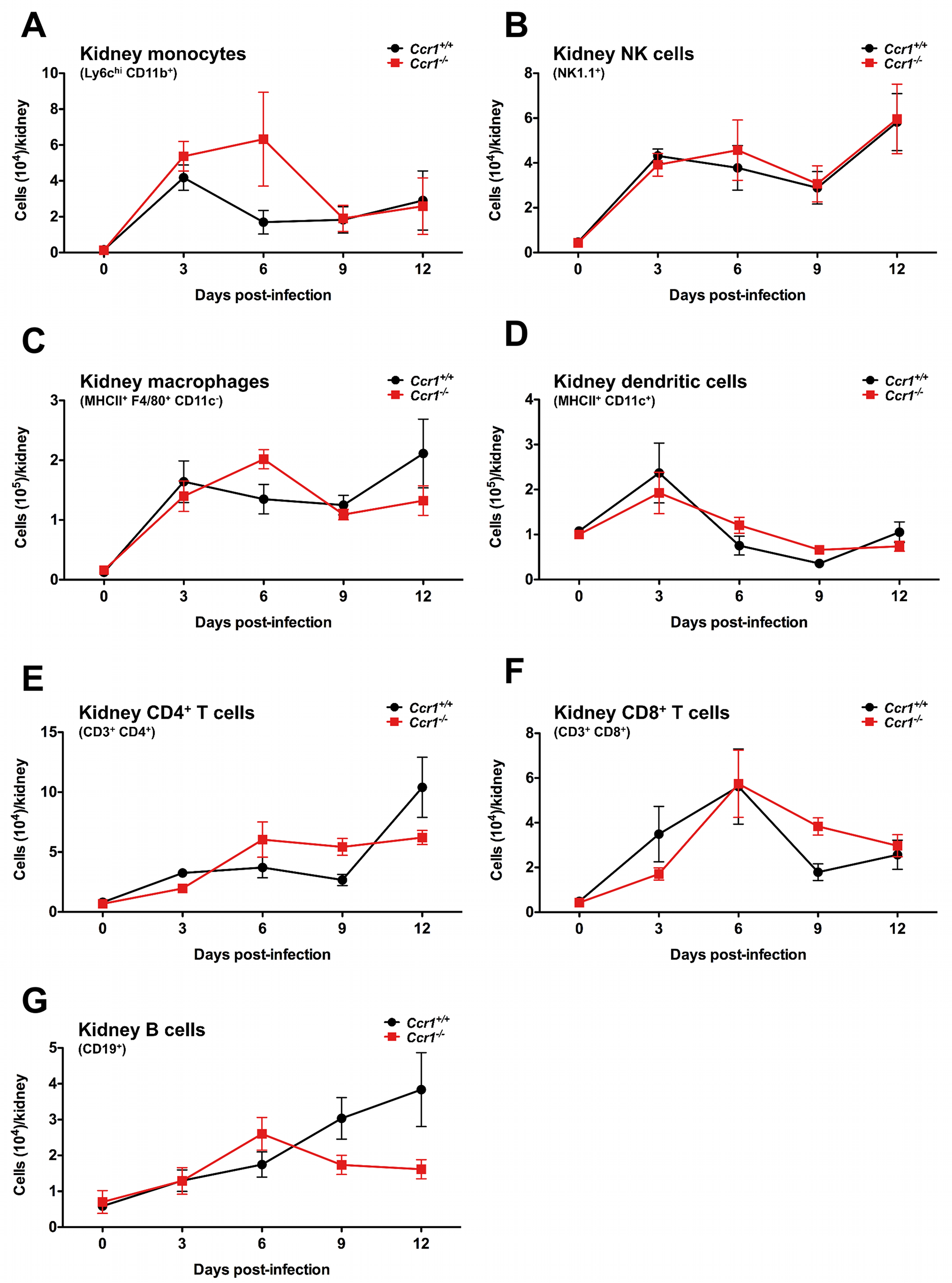

Supplement: Figure S3 — Ccr1 deficiency only affects accumulation of neutrophils in the kidney in a mouse model of invasive candidiasis. Accumulation is shown for (A) inflammatory monocytes, (B) NK cells, (C) macrophages, (D) dendritic cells, (E) CD4+ T cells, (F) CD8+ T cells, and (G) B cells in the kidney post-Candida infection. Data are from two to four independent experiments using six to twelve Ccr1+/+ and six to twelve Ccr1−/− mice per time-point. (TIF) [file ppat.1002865.s003.tif]

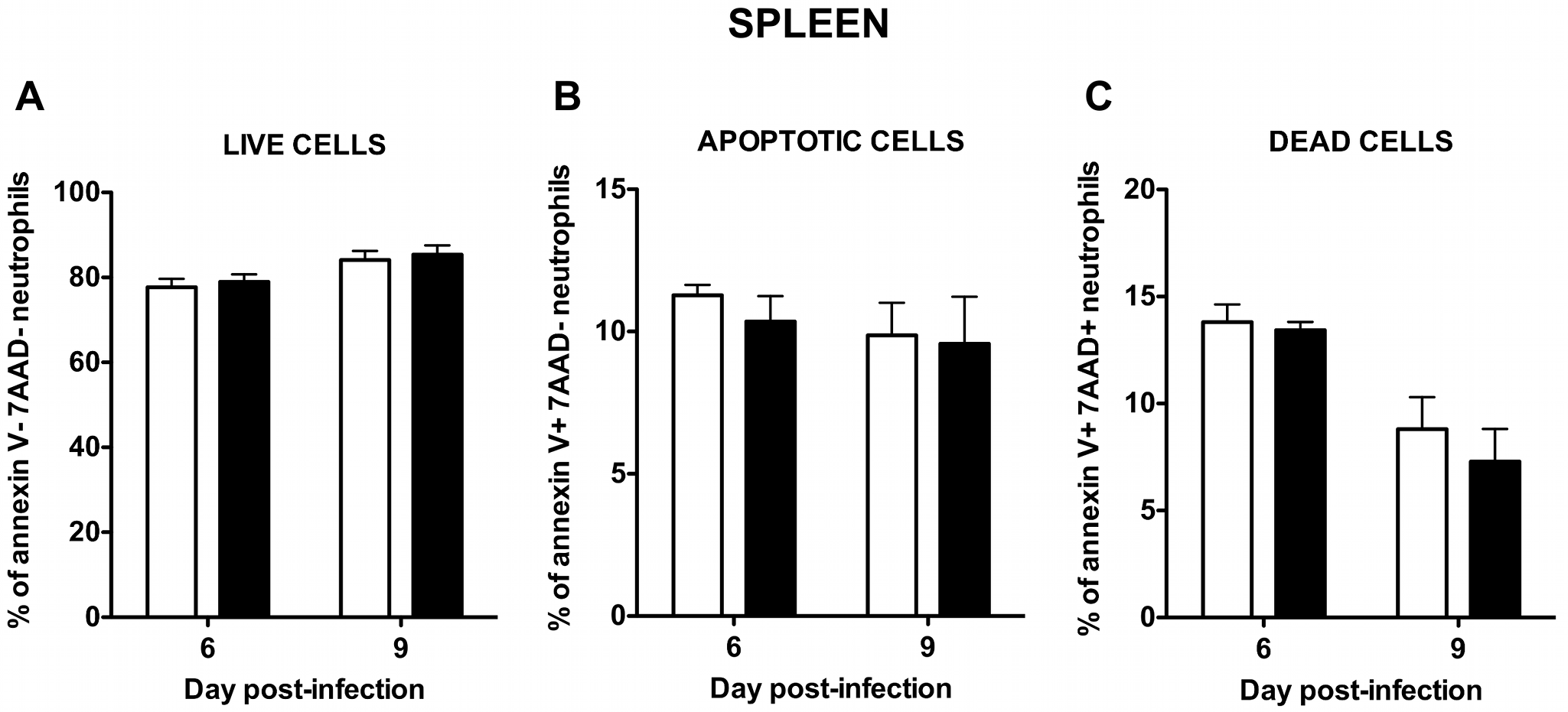

Supplement: Figure S4 — Ccr1 deficiency does not affect survival of neutrophils in the spleen after Candida infection. Percent of (A) live annexin V− 7-AAD−, (B) apoptotic annexin V+ 7-AAD−, and (C) dead annexin V+ 7-AAD+ splenic neutrophils is similar in Ccr1+/+ and Ccr1−/− mice at days 6 and 9 post-infection. Data are shown from one of two independent experiments with similar pattern of results using a total of seven Ccr1+/+ and seven Ccr1−/− mice per time-point. (TIF) [file ppat.1002865.s004.tif]

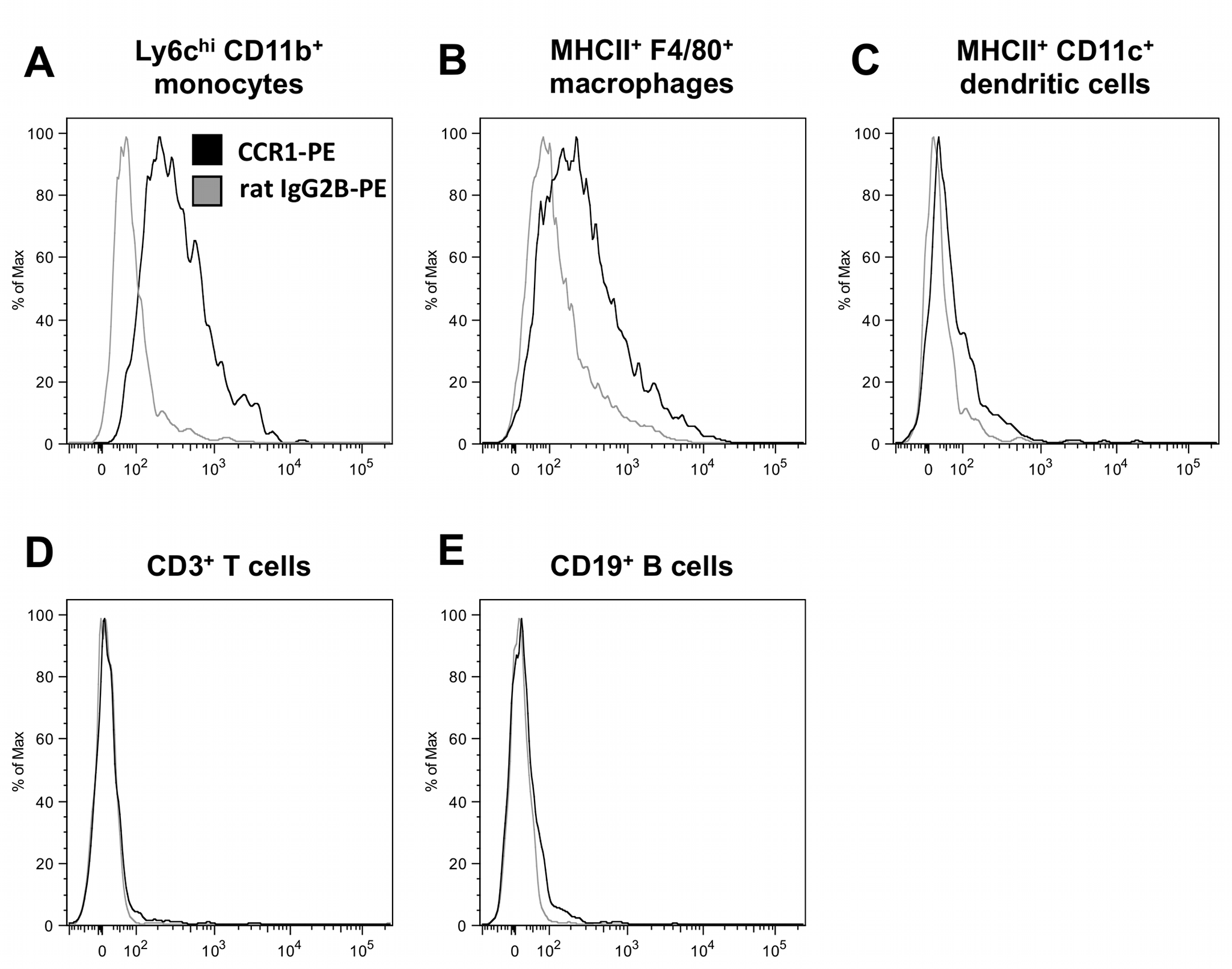

Supplement: Figure S5 — Ccr1 expression on leukocyte subsets in the kidney in a mouse model of invasive candidiasis. (A) monocytes, (B) macrophages, (C) dendritic cells, (D) T cells and (E) B cells. Data are from day 6 post-infection and are representative FACS histograms from two independent experiments using four to six Ccr1+/+ and four to six Ccr1−/− mice per time-point. (TIF) [file ppat.1002865.s005.tif]

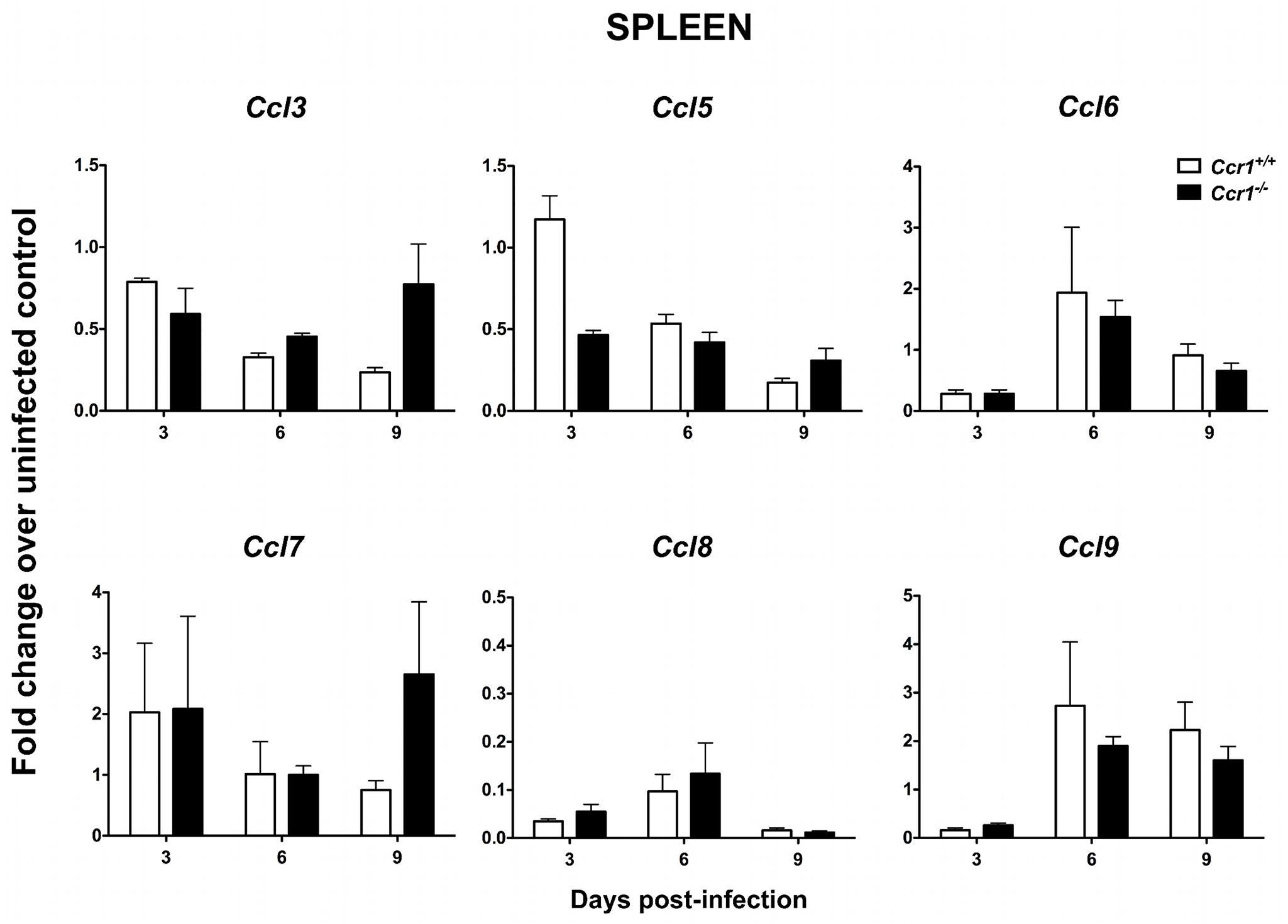

Supplement: Figure S6 — Ccr1 deficiency does not affect expression of Ccr1 ligands in the spleen in a mouse model of invasive candidiasis. Data are from one experiment using four Ccr1+/+ and four Ccr1−/− mice per time-point. (TIF) [file ppat.1002865.s006.tif]

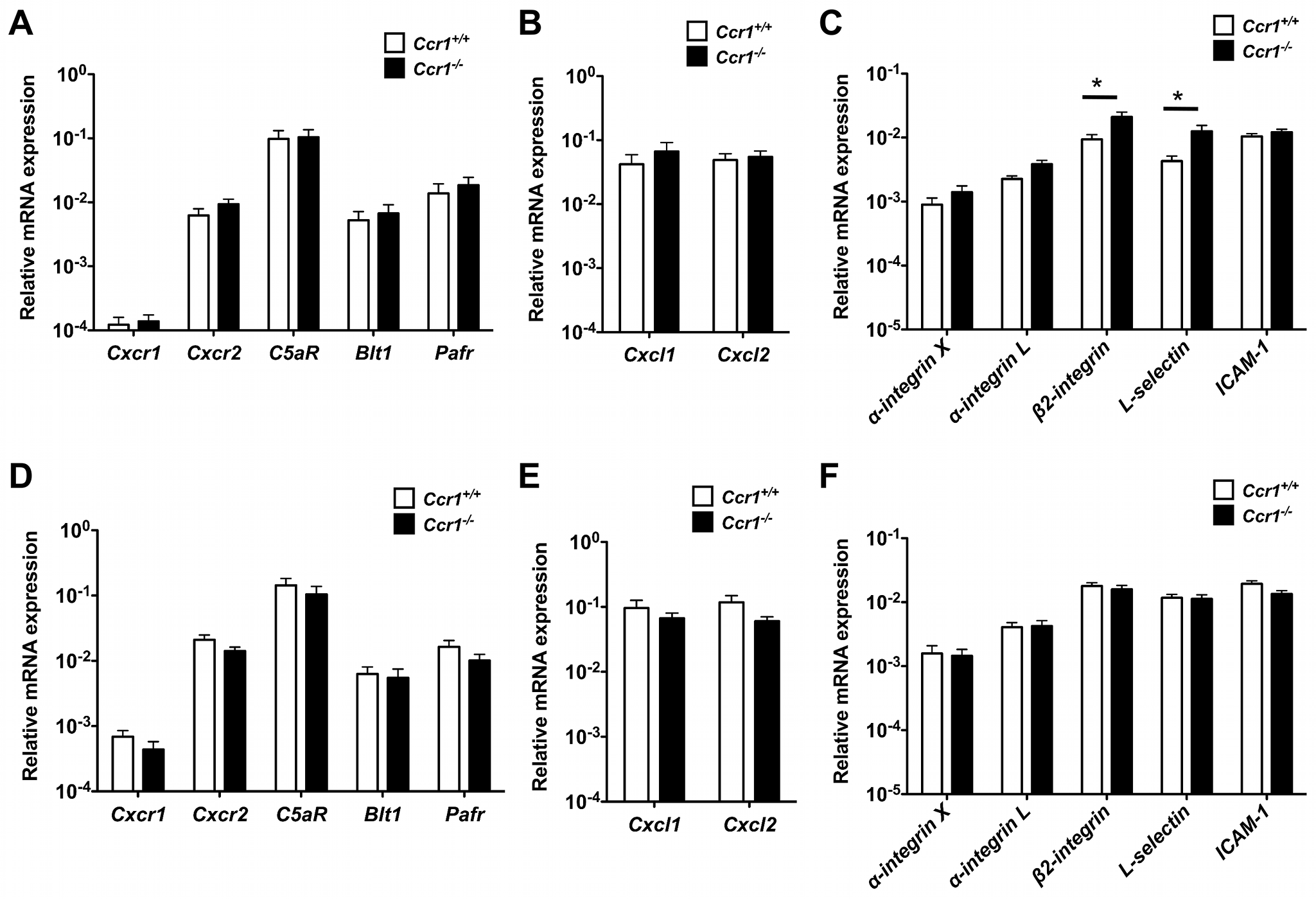

Supplement: Figure S7 — Ccr1 deficiency does not decrease the expression of other neutrophil-targeted chemotactic factors or adhesion molecules in Candida -infected kidneys. Relative mRNA expression is shown for the indicated factors in Ccr1+/+ and Ccr1−/− kidneys at days 3 (A–C) and 6 (D–F) after Candida infection. Data are from two independent experiments using seven Ccr1+/+ and seven Ccr1−/− mice per time-point. * P = 0.02. (TIF) [file ppat.1002865.s007.tif]

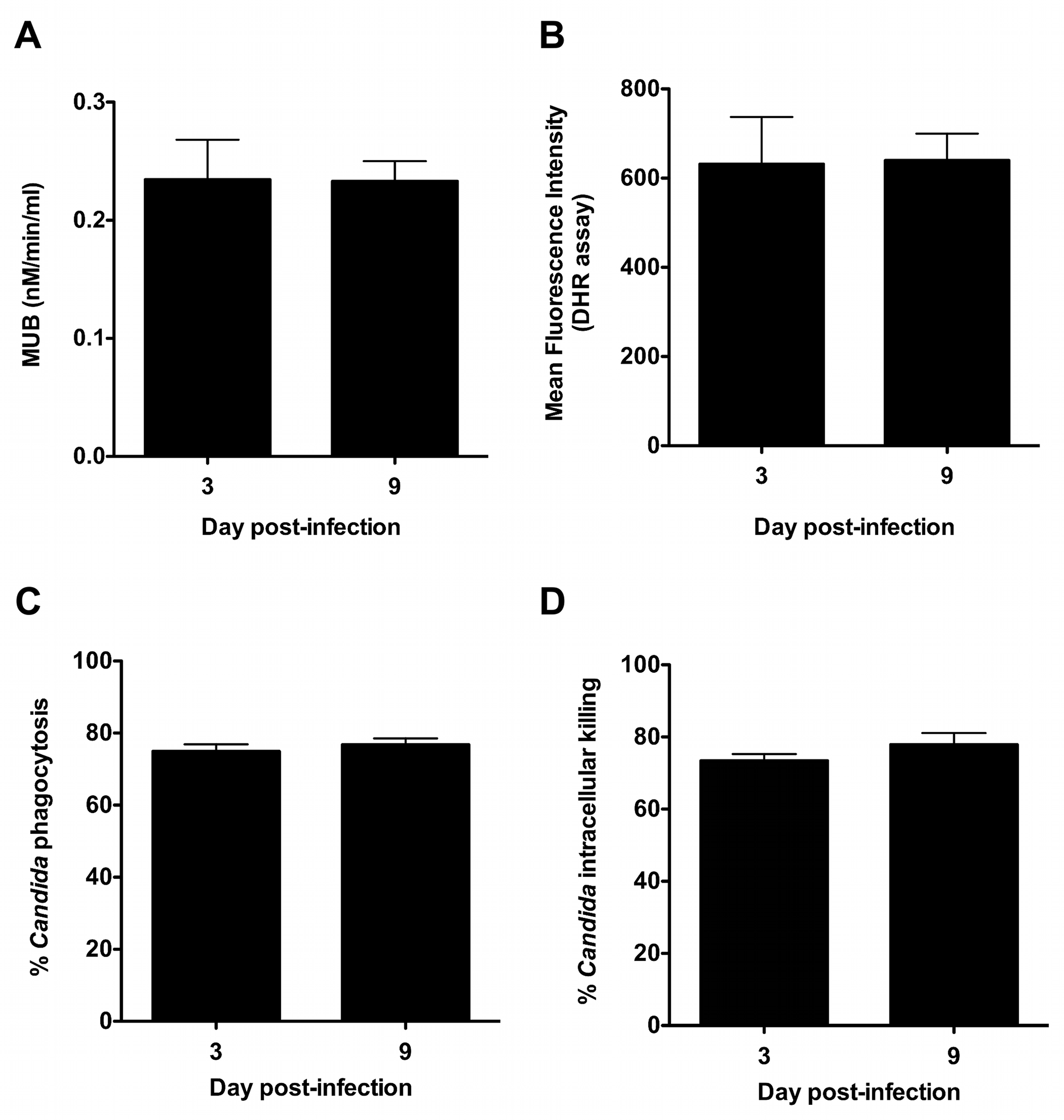

Supplement: Figure S8 — Ccr1 expression on kidney neutrophils does not significantly affect their immunopathogenic potential or anti- Candida effector function. Sorted Ccr1lo (day 3) and Ccr1high (day 9) kidney neutrophils do not differ in their capacity for degranulation (A), oxidative burst (B), Candida phagocytosis (C) or anti-Candida killing (D). Data are from two independent experiments using 6–10 Ccr1+/+ mice per time-point for degranulation and oxidative burst assays, 7–10 Ccr1+/+ mice per time-point for phagocytosis assays, and 8–10 Ccr1+/+ mice per time-point for killing assays. (TIF) [file ppat.1002865.s008.tif]
