# Supplementary material for: Chemokine Receptor Ccr1 Drives Neutrophil-Mediated Kidney Immunopathology and Mortality in Invasive Candidiasis
Source: PLoS Pathog. 2012 Aug 16;8(8):e1002865. doi: 10.1371/journal.ppat.1002865 (PMC3420964; doi:10.1371/journal.ppat.1002865)
Supplement: Table S1 — Sequences of the primers used for qPCR with SYBR Green in the present study. (PDF) [file ppat.1002865.s009.pdf]

**Gene product**	**Primer name**[*]	**Primer sequence (5' → 3')**	**Amplicon length (bp)**
Gapdh	Gapdh F	aactttggcattgtggaagg	223
	Gapdh R	acacattgggggtaggaaca	
Ccr1	Ccr1 F	gttgggaccttgaaccttga	250
	Ccr1 R	cccaaaggctcttacagcag	
Ccr2	Ccr2 F	agagagctgcagcaaaaagg	185
	Ccr2 R	ggaaagaggcagttgcaaag	
Ccr3	Ccr3 F	tttcctgcagtcctcgctat	181
	Ccr3 R	ataagacggatggccttgtg	
Ccr4	Ccr4 F	gctcctcttacacgcagtcc	180
	Ccr4 R	cttgccatggtcttggtttt	
Ccr5	Ccr5 F	cgaaaacacatggtcaaacg	176
	Ccr5 R	ttcctactcccaagctgcat	
Ccr6	Ccr6 F	ttgtcctcaccctaccgttc	239
	Ccr6 R	gatgaaccacactgccacac	
Ccr7	Ccr7 F	gtgtgcttctgccaagatga	154
	Ccr7 R	ccacgaagcagatgacagaa	
Ccr9	Ccr9 F	cttgccactcttcccttctg	170
	Ccr9 R	gccttcatggcctgtacaat	
Ccr10	Ccr10 F	tgccatctcaggcctctact	160
	Ccr10 R	cagccacacgaagactgaaa	
Cxcr1	Cxcr1 F	ccgtcatggatgtctacgtg	170

	Cxcr1 R	cagcagcaggataccactga	
Cxcr2	Cxcr2 F	ggtggggagttcgtgtagaa	201
	Cxcr2 R	cgaggtgctaggatttgagc	
Cxcr3	Cxcr3 F	tgctagatgcctcggacttt	214
	Cxcr3 R	cgctgactcagtagcacagc	
Cxcr4	Cxcr4 F	tcagtggctgacctcctctt	220
	Cxcr4 R	tttcagccagcagtttcctt	
Cxcr5	Cxcr5 F	atggccttaatgtgcctgtc	188
	Cxcr5 R	cttctggaacttgccctcag	
Cxcr6	Cxcr6 F	tagtggctgtgttcctgctg	219
	Cxcr6 R	ggcagccgatatccttcata	
Cxcr7	Cxcr7 F	ggtcagtctcgtgcagcata	153
	Cxcr7 R	gtgccggtgaagtaggtgat	
Xcr1	Xcr1 F	agcagccactgtcttgacct	153
	Xcr1 R	ggcatgtggaagacaacctt	
Cx3cr1	Cx3cr1 F	ggagactggagccaacagag	196
	Cx3cr1 R	tcttgtctggctgtgtcctg	
D6	D6 F	cctgcttctcattgtcatggt	150
	D6 R	cgcaggtacagcttccaaa	
Duffy	Duffy F	caaggggctgaagatagcac	190
	Duffy R	ggcttctgtcacattcagca	
CCX CKR	CCX CKR F	gcagttcgcaaaagtcttcc	176
	CCX CKR R	aaggcagcgtgatcagaagt	

Ccl6	Ccl6 F	ggctttggaatgtgtctggt	215
	Ccl6 R	ctggccccgtagttctatga	
Ccl7	Ccl7 F	gcatggaagtctgtgctgaa	157
	Ccl7 R	agaaagaacagcggtgagga	
Ccl9	Ccl9 F	agtggtctgtgggactttgg	194
	Ccl9 R	cagacctgtggctgcataga	
Ccl11	Ccl11 F	tccacagcgcttctattcct	178
	Ccl11 R	ctatggctttcagggtgcat	
Ccl12	Ccl12 F	gggaagctgtgatcttcagg	177
	Ccl12 R	gggaacttcaggggga aata	
Ccl17	Ccl17 F	agtggagtgttccagggatg	193
	Ccl17 R	ctggtcacaggccgttttat	
Ccl19	Ccl19 F	caagaacaaaggcaacagca	236
	Ccl19 R	cggctttattggaagctctg	
Ccl20	Ccl20 F	cgactgttgcctctcgtaca	177
	Ccl20 R	aggaggttcacagccctttt	
Ccl24	Ccl24 F	ggcaatagcaccgaggttta	215
	Ccl24 R	cagcacaggagacaccagaa	
Ccl25	Ccl25 F	gggaatccagaggacatgaa	248
	Ccl25 R	cctccagctggtgcttactc	
Ccl27	Ccl27 F	atagacagccactcccaagc	187
	Ccl27 R	acagtcccttggagcctttt	
Ccl28	Ccl28 F	tggcaaaagccacattcata	194
	Ccl28 R	catgccagagtcgaacagaa	

Cxcl1	Cxcl1 F	gctgggattcacctcaagaa	180
	Cxcl1 R	tctccgttacttggggacac	
Cxcl2	Cxcl2 F	aagtttgccttgaccctgaa	180
	Cxcl2 R	aggcacatcaggtacgatcc	
Cxcl4	Cxcl4 F	agtcctgagctgctgcttct	226
	Cxcl4 R	ggcaaattttcctcccattc	
Cxcl5	Cxcl5 F	gaaagctaagcggaatgcac	166
	Cxcl5 R	gggacaatggtttccctttt	
Cxcl7	Cxcl7 F	gcgctgcagatgtacgaata	226
	Cxcl7 R	aggaaaatggtttggcacag	
Cxcl9	Cxcl9 F	aaaatttcatcacgcccttg	207
	Cxcl9 R	tctccagcttggtgaggtct	
Cxcl10	Cxcl10 F	gccgtcattttctgcctcat	127
	Cxcl10 R	gcttccctatggccctcatt	
Cxcl11	Cxcl11 F	agtaacggctgcgacaaagt	225
	Cxcl11 R	gcatgttccaagacagcaga	
Cxcl12A	Cxcl12A F	cttcatccccattctcctca	171
	Cxcl12A R	gactctgctctggtggaagg	
Cxcl12B	Cxcl12B F	agtagtggctccccaggttt	250
	Cxcl12B R	gagacagtcttgcggacaca	
Cxcl13	Cxcl13 F	catcatgaggtggtgcaaag	188
	Cxcl13 R	gggtcacagtgcaaaggaat	
Cxcl14	Cxcl14 F	ctgcgcaccagacttcatta	271
	Cxcl14 R	gaccagtcataagcccagga	

Cxcl16	Cxcl16 F	gggaagagttttcaccacca	167
	Cxcl16 R	ggttgggtgtgctctttgtt	
Xcl1	Xcl1 F	cagggccagtaccagaaaga	150
	Xcl1 R	caatgggtttgggaactgag	
Cx3cl1	Cx3cl1 F	ggaaagaaacgtggtccaga	165
	Cx3cl1 R	gccctcagaatcacagggta	
C5aR	C5aR F	caagacgctcaaagtggtga	244
	C5aR R	tatgatgctggggagagacc	
Blt1	Blt1 F	ttgctcactgctcccttttt	237
	Blt1 R	aaaagacaccacccagatgc	
Pafr	Pafr F	agcagagttgggctaccaga	164
	Pafr R	tgcgcatgctgtaaaacttc	

* F, forward; R, reverse; C5ar, complement C5a receptor; Blt1, leukotriene B4 receptor 1; Pafr, platelet activating factor receptor; ICAM-1, intercellular adhesion molecule 1.

Taqman primers and probes for Ccr8, Blt2, Ccl1, Ccl2, Ccl3, Ccl4, Ccl5, Ccl8, Ccl11, Ccl21, Ccl22, Ccl26, Cxcl3, Cxcl15, Cxcl17, α-integrin X, α-integrin L, β2-integrin, L-selectin and ICAM-1 were obtained from Applied Biosystems and their sequences are not available.
